# High-resolution fMRI reveals a dorsal brain pathway selective for conspecific vocalizations in macaques

**DOI:** 10.1162/IMAG.a.108

**Published:** 2025-08-13

**Authors:** Mathilda Froesel, Koh Ikuchi, Qi Zhu, Haiyan Wang, Marc Hauser, Suliann Ben Hamed, Wim Vanduffel

**Affiliations:** Department of Neurosciences, Lab for Neuro- and Psychophysiology, KU Leuven, Leuven, Belgium; Leuven Brain Institute, Leuven, Belgium; Division of Physiology and Neurobiology, Department of Neuroscience, Graduate School of Medicine, Kyoto University, Japan, Kyoto, Japan; Cognitive Neuroimaging Unit, INSERM, CEA, Université Paris-Saclay, NeuroSpin Center, Gif/Yvette, France; Brainnetome Center, Institute of Automation, Chinese Academy of Sciences, Beijing, China; Risk-Eraser, West Falmouth, MA, United States; Institut des Sciences Cognitives Marc Jeannerod, Bron Cedex, France; Martinos Center for Biomedical Imaging and Department of Radiology, Harvard Medical School, Boston, MA, United States

**Keywords:** macaques, vocalizations, fMRI, dorsal premotor cortex, parieto-temporal cortex

## Abstract

Understanding the neural basis of social communication and vocal perception in primates is a key challenge in systems neuroscience. Vocalizations are fundamental for communication, and several cortical areas, known as “voice patches,” have been identified as being sensitive to conspecific vocalizations in primates, vital for distinguishing species-specific calls. While the dorsal stream’s role in complex auditory-motor functions and human speech processing is established, its specific contribution to processing species-specific vocalizations in non-human primates remains unclear. Using high-resolution fMRI (0.6 mm isotropic voxels), we investigated brain regions involved in processing vocalizations in awake rhesus monkeys exposed to coos, screams, and aggressive calls, among control sounds. Our analyses revealed a widespread network involved in vocalization processing, encompassing auditory-associated areas including the core and belt auditory cortices, as well as premotor, and somatosensory-related areas. Moreover, we found selective activation in the caudal part of the lateral sulcus (area Tpt) and the dorsal premotor cortex (area 6DR/F2) in response to vocalizations. Also, the population responses in these areas could discriminate between different vocalizations. Our results enhance our understanding of the neural basis of vocal communication in primates. Specifically, they highlight the involvement of a voice-related dorsal network in macaques, including Tpt and part of 6DR/F2, in processing the acoustic features of salient vocal stimuli, potentially linking them to motor representations. These findings provide insights into potential evolutionary precursors of auditory-motor pathways that support complex auditory communication systems in primates, including human speech.

## 1 Introduction

The investigation of auditory perception and its neural foundations has attracted significant attention (Banno et al., 2020; Cohen, 2013). Anatomical tract tracing studies indicate that the anterior (rostral) belt of auditory cortex in the lateral sulcus connects to the ventrolateral prefrontal cortex (vLPFC), while the posterior (caudal) auditory cortex links to the dorsolateral prefrontal cortex (Romanski et al., 1999; Romanski & Averbeck, 2009). Functionally, the rostral lateral auditory belt is associated with sound identification, particularly vocalizations, whereas the caudal lateral auditory belt is linked to sound localization (Kuśmierek & Rauschecker, 2014; Recanzone et al., 2000; Tian et al., 2001). Initially, the auditory system was characterized by ventral and dorsal pathways, analogous to the visual system (Goodale & Milner, 1992; Mishkin et al., 1983), with the ventral stream processing the ‘what’ and the dorsal stream the ‘where’ aspects of perceived sounds (Rauschecker, 1998; Rauschecker & Tian, 2000). However, subsequent research has proposed that the dorsal auditory pathway, particularly the postero-dorsal stream, is involved in speech and language processing in humans, functioning as a sensorimotor interface—a ‘how’ pathway—bridging sensory and motor networks (Rauschecker, 2011; Rauschecker & Scott, 2009).

Auditory perception is essential for social cognition. Macaques use vocalizations to communicate important information about their environment, mood, and feelings with their conspecifics (Gouzoules et al., 1984; Hauser, 1991; Hauser & Marler, 1993). Although these vocalizations are not directly comparable with human speech in complexity or articulatory control, they are crucial for social communication, conveying important information that is perceived and contextually interpreted by conspecifics, enabling appropriate social responses. Understanding the neural networks supporting vocal perception and interpretation in macaques may offer insights into the evolutionary precursors or homologous brain regions involved in complex auditory communication systems like human vocal communications (Belin et al., 2018).

Anatomical studies in primates, including macaques and humans, have identified major white matter tracts, such as the superior longitudinal fasciculus/arcuate fasciculus (SLF/AF) complex. The arcuate fasciculus connects posterior temporal regions, as area Tpt in macaques (analogous to caudal STG in humans), and various frontal cortical areas, including parts of dorsal and ventral premotor cortex (areas 6), prefrontal cortex (areas 8, 46), and areas 44 and 45 (Becker et al., 2022; Petrides & Pandya, 2009; Romanski et al., 1999; Romanski & Averbeck, 2009; Schmahmann & Pandya, 2006). In humans, these anatomically connected temporal and frontal regions, linked by the AF and SLF, form critical pathways supporting language, with the dorsal stream being prominently implicated in sensorimotor integration, such as mapping sounds to articulation (Hickok & Poeppel, 2004; Saur et al., 2008).

Research employing diverse methods with humans, macaques (Old world monkeys), and marmosets (New world monkeys) has uncovered regions known as “voice patches”, which seem to be selectively activated by conspecific vocalizations (see for review Belin et al., 2018). In macaques, functional magnetic resonance imaging (fMRI) has revealed a voice-specific region in the anterior superior temporal gyrus (STG) that plays a role in identity coding and discrimination based on voice (Perrodin et al., 2014; Petkov et al., 2008). This anterior temporal voice area (TVa) contains neurons that demonstrate voice selectivity (Perrodin et al., 2011). Other areas involved in voice processing have also been noted. Electrophysiological recordings showed that neurons of vlPFC (areas 12 and 45) encode information about different types of vocalizations (Romanski et al., 2005). PET studies revealed complementary parts of this vocalization-processing network, including a high-level auditory area corresponding to the cytoarchitectonic temporoparietal transition area Tpt (Gil-da-Costa et al., 2006). This posterior peri-sylvian area, however, is mentioned infrequently in recent literature, as it is considered more posterior than the auditory-processing areas typically described (Joly, Pallier, et al., 2012). Furthermore, recent fMRI work in marmosets suggests that dorsal premotor regions (analogous to macaque 6DR) are involved in vocalization processing even during passive listening (Dureux et al., 2024; Jafari et al., 2023). While area 6DR/F2 has been implicated in motor control and auditory-motor learning in macaques (e.g., Archakov et al., 2020; Rauschecker & Scott, 2009), its specific functional contribution and representational properties in macaque vocalization perception within this dorsal stream framework has not been characterized by fMRI.

Building on this established anatomical framework in primates and the evidence for vocalization processing in interconnected temporal and frontal regions, our study aimed to further characterize the functional role of key areas within this network in macaques. Despite the known anatomical connections between these temporal and frontal regions in primates, as well as emerging evidence of their involvement in auditory and vocal processing, the precise functional properties and representational content of especially dorsal regions during macaque vocal perception remain unclear. Addressing these gaps, our study had two main objectives. First, utilizing high-resolution fMRI with implanted phased-array receive coils to enhance signal detection in these areas, we aimed to provide a detailed description of the extensive network responsive to macaque vocalizations, with a specific focus on the involvement and functional properties of dorsal vocal processing regions. Second, we sought to determine whether different types of vocalizations from the macaque repertoire—such as affiliative coos, submissive screams and antagonistic aggressive calls—elicit distinct neural responses and representations within this network, characterizing the selectivity and discriminability of responses using fMRI signal change and multivoxel pattern analysis.

## 2 Materials and Methods

### 2.1 Subjects

Two adult rhesus monkeys (Macaca mulatta), one female (M50; 6 kg; 13 years old) and one male (M58; 9.6 kg; 7 years old) participated in the experiment. These animals were raised in captivity, experiencing social interactions primarily with their own species in group housing and with humans during experiments and maintenance of the facilities. All animals were held at the primate facility of the KU Leuven Medical School.

### 2.2 Ethical statement

The animals were group-housed in cages sized 16–32 m^3^, which encourages social interactions and locomotor behavior. The environment was enriched by foraging devices and toys. The animals were fed daily with standard primate chow supplemented with fruits, vegetables, bread, peanuts, cashew nuts, raisins, and dry apricots. The animals were water restricted but could work every day until satiated. During non-working days, they received water in their living quarters. Animal care and experimental procedures met the National Institute of Health’s Guide for the Care and Use of Laboratory Animal guidelines, the European legislation (Directive 2010/63/EU) and were approved by the Animal Ethics Committee of KU Leuven. Weatherall reports were used as reference for animal housing and handling.

Before the scanning sessions, monkeys were trained daily to perform a visual fixation task with the head rigidly fixed to a primate chair. The fixation task was used to minimize body movement during scanning and to equalize attention across conditions. Details concerning head-post surgery and behavioral procedures are described in (Vanduffel et al., 2001).

### 2.3 Experimental design

**Fig. 1.**
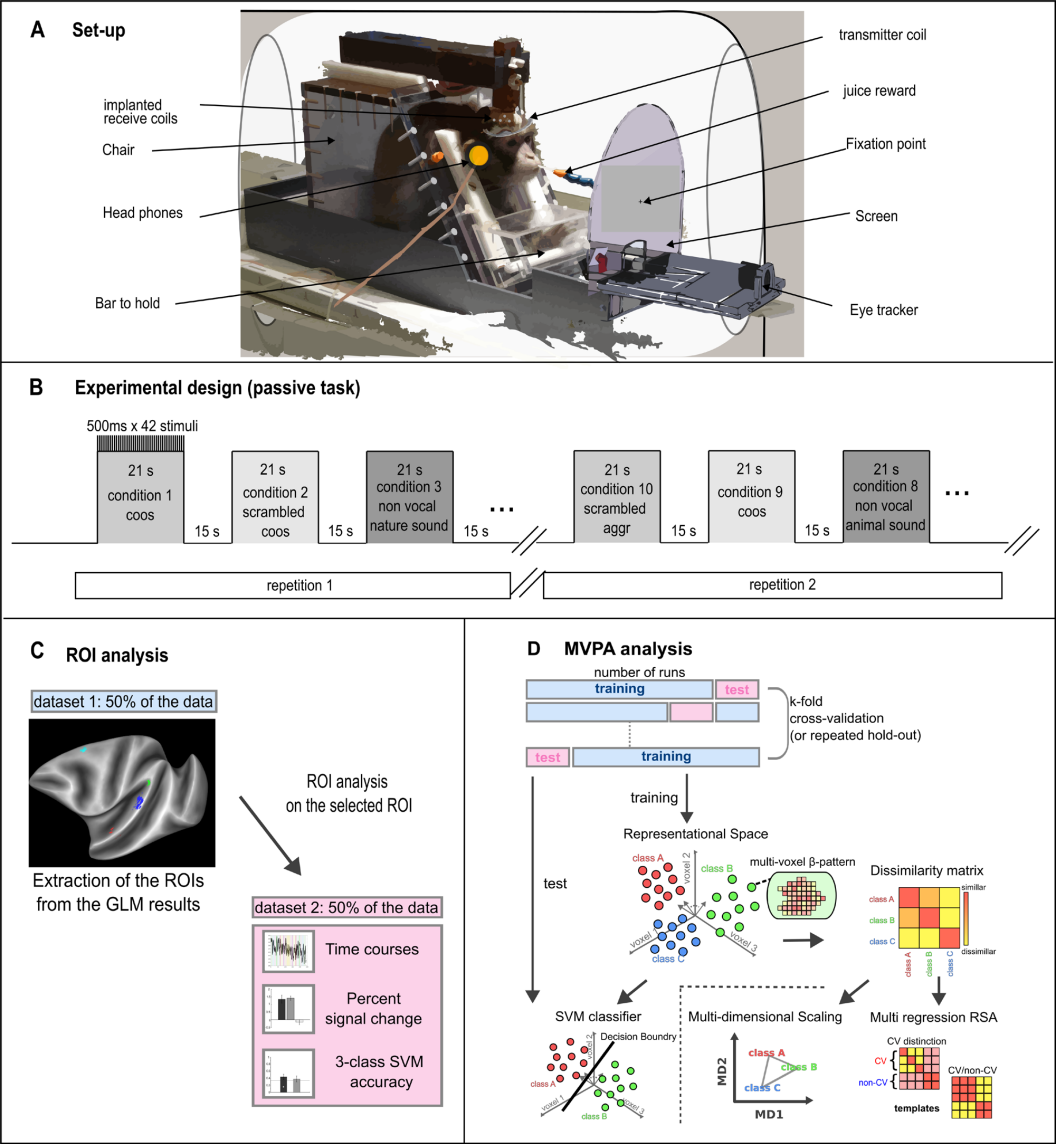
Experimental design and analysis. (A) Set-up. (B) Experimental design: Blocks (21 s) of auditory stimuli of 5 categories and their scrambled counterparts, 10 types of blocks in total. Every block contained 42 stimuli presented for 500 ms. The different blocks are presented randomly two times in a run and are separated by silent blocks (15 s). (C) Explanation of types of ROI analyses. Half of the data was used to extract the ROIs from the GLM, and the ROI analysis was performed on the other half. (D) MVPA analysis types. Multivariate pattern classification analysis (MVPC) was performed using support vector machine (SVM) analyses. Separately for each monkey and each region of interest (ROI), an SVM classifier was trained on β-patterns responding to each sound category from (NRuns – 1) runs, and then tested on data from the held-out run. A dissimilarity matrix (DSM) was computed to visualize the representational geometry of different sound categories within each ROI. To visualize the DSM for each ROI, we performed non-classical multi-dimensional scaling with the matrix and projected the dissimilarity relationship onto a two-dimensional space.

In the MR scanner, the monkeys performed a passive fixation task. To minimize movement, they were instructed to fixate on a central cross projected onto a translucent screen located at 57 cm from their eyes using a Barco LCD projector (60 Hz refresh rate, 1400 x 1050 resolution). They were also required to keep their two hands still on response buttons (see Fig. 1A), but there was no behavioral response to the sounds. The auditory stimuli were presented at ~80 dB sound pressure level (SPL) through MR–compatible headphones (Baumgart et al., 1998), customized for monkeys (MR Confon GmbH, Magdeburg, Germany), which reduced scanner noise to below 73 dB SPL (see Joly, Ramus, et al., 2012). Eye movements were monitored using an eye tracking system (ISCAN, 120 Hz), and only runs in which fixation was maintained within a 2 × 2 degree window for more than 90% of the time, and the hands were kept still on the response buttons, were included in the analysis.

The experiment employed a block design with ten different categories of sound, each presented twice within a run (1: coos, 2: scrambled coos, 3: nature, 4: scrambled nature, 5: screams, 6: scrambled screams, 7: animal, 8: scrambled animal, 9: aggressive calls, 10: scrambled aggressive calls). Each block lasted 21s, during which 42 stimuli of the same category were repeated every 500 ms. Each sound block was followed by a block of 15 s silences (see Fig. 1B).

The blocks were counterbalanced across runs using a Latin square randomization, ensuring each condition appeared once in each serial position.

### 2.4 Stimuli

Five sound categories were employed in this experiment: three types of macaque vocalizations (coos, aggressive calls, and screams), as well as animal calls (bird, dog, horse, chimpanzee, pig, lion) and nature sounds (storm, wind, fire). The macaque vocalizations were sourced from the *Rhesus Monkey Repertoire* database provided by Marc Hauser, with recordings collected over several years in Cayo Santiago, Puerto Rico. We selected ten stimuli produced by various individuals of both sexes, encompassing three social types of social calls, one with positive social valence (coos), and two with negative social valence (aggressive calls and screams) (Gouzoules et al., 1984; Hauser & Marler, 1993). The animal and nature sound stimuli came from the dataset https://doi.org/10.5061/dryad.np4hs already used in Erb et al. (2019).

No attempt was made to equate the acoustic structure across categories, leading to natural systematic differences that might influence both perceptual and neural responses. Like Kuśmierek and Rauschecker (2009), non-vocalizations were, on average, longer (mean duration: 1.00 s for nature and animal calls vs. 0.51 s for vocalizations) (Kuśmierek & Rauschecker, 2009). In the experiment, all stimuli were truncated or padded to a 0.5-s duration. Vocalizations were presented in their entirety, while some non-vocal sounds were shortened. Care was taken to ensure that truncated sounds remained identifiable as complete acoustic events.

**Fig. 2.**
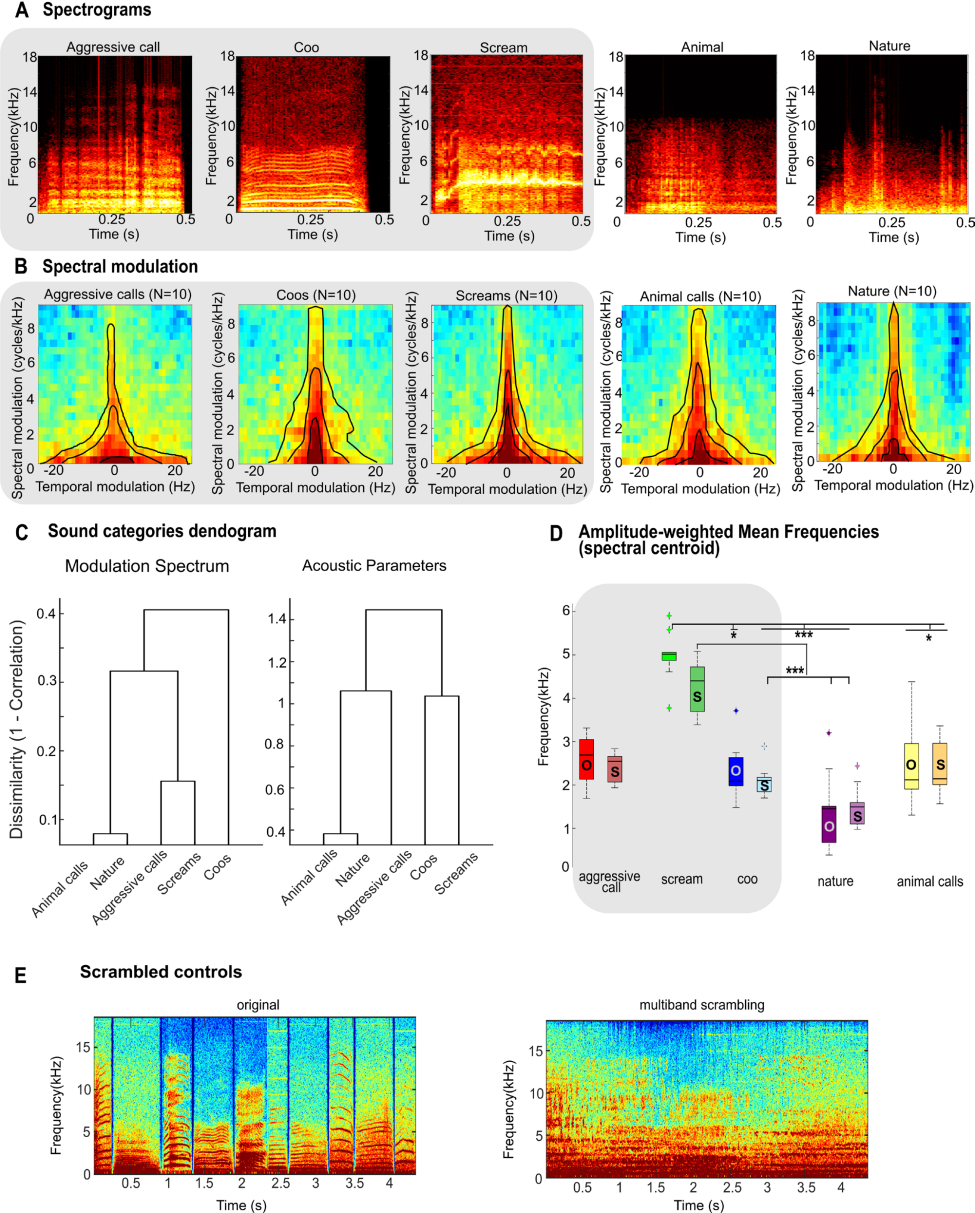
Acoustic characterization of sound categories. (A) Example auditory spectrograms for each of the five sound categories. (B) Average modulation power spectra (MPS; N = 10 sounds per category). Axes represent temporal modulation rate (Hz) and spectral modulation scale (cycles/kHz). Color intensity indicates average log power (dB, arbitrary units referenced to the peak), with warmer colors indicating higher power. Black contour lines encircle the half-maximum of the MPS. (C) Hierarchical clustering dendrograms illustrating acoustic dissimilarity. Dissimilarity (y-axis; correlation distance: 1−Pearson’s r) is based on (left) vectorized average MPS and (right) acoustic features (see Table 1; Methods for details). (D) Spectral centroids (amplitude-weighted mean frequencies, kHz) for each sound category. Box plots show median, interquartile range, and whiskers extending to 1.5 x IQR. Asterisks denote significance levels from post-hoc pairwise comparisons (Bonferroni corrected: **p < 0.01, ***p < 0.001). “O” = original sound, and “S” = scrambled version. (E) Example spectrograms illustrating the acoustic scrambling procedure: (left) a sequence of original sounds and (right) its multiband scrambled version, which preserves long-term spectral characteristics but disrupts temporal fine structure. Macaque vocalizations (Aggressive call, Coo, Scream) are distinguished by grey backgrounds for their respective labels in (A) and (B), and for their corresponding plots in (D).

The acoustic characteristics of the sound categories are detailed in Figure 2 and Table 1. Example auditory spectrograms (Fig. 2A) and average modulation power spectra (MPS; Fig. 2B) illustrate key spectro-temporal features. The MPS for each stimulus was derived from its auditory spectrogram by computing the 2D Fast Fourier Transform (FFT) of the spectrogram envelope’s autocorrelation across frequency bands. Average MPS were then calculated for each category (Kuśmierek et al., 2012; Singh & Theunissen, 2003). The MPS analysis (Fig. 2B) revealed that non-vocalizations exhibited a broader energy distribution along the temporal modulation axis compared to vocalizations. This was corroborated by a higher spectral spread (power spectrum standard deviation, PSSD), with a mean PSSD of 20.8 Hz for non-vocalizations versus 15.56 Hz for vocalizations, suggesting greater frequency dispersion in nature and animal sounds. Conversely, spectral variability, as measured by the power spectrum coefficient of variation (PSCV), was higher in vocalizations (mean PSCV: 5.4) compared to non-vocalizations (mean PSCV: 2.7). The differences in PSSD and PSCV between vocalization and non-vocalizations were driven mainly by coos and aggressive calls, with screams showing an intermediate PSCV and a PSSD that were closer to those of non-vocalizations. Vocalizations were generally more periodic, with coos exhibiting the highest harmonic-to-noise ratio (HNR; mean: 0.83), followed by screams (0.67). Animal calls were similar to these two vocalization types (0.64). Nature sounds (0.37) and aggressive calls (0.39) displayed lower HNR values, reflecting their noisier or more aperiodic structure (see Table 1 for specific values for each category).

**Table 1.** Mean acoustic parameters for sound categories.

Sound category	RMS amplitude (dB)	Mean F0 (Hz)	F0 SD	HNR	PSSD	PSCV	Mean duration (s)	Spectral centroid(kHz)
**Aggressive calls**	**-14.9**	**430**	**252**	**0.39**	**15.72**	**5.5**	**0.25**	**2.69**
**coos**	**-9.95**	**581**	**54**	**0.83**	**13.59**	**6.8**	**0.4**	**2.08**
**screams**	**-13.06**	**684**	**224**	**0.67**	**17.36**	**2.17**	**0.8**	**5.01**
Animal calls	-23.45	503	203	0.64	20.76	3.77	1	2.11
Nature sounds	-23.57	439	264	0.37	20.84	1.63	1	1.45

Values represent the mean for each acoustic feature across the N = 10 stimuli within each of the five sound categories. Macaque vocalizations (Aggressive call, Coo, Scream) are distinguished with bold.

Abbreviations: RMS, Root Mean Square amplitude; F0, Fundamental frequency; F0 SD, Standard Deviation of F0; HNR, Harmonic-to-Noise Ratio; PS SD, Power Spectrum Standard Deviation (spectral spread); PS CV, Power Spectrum Coefficient of Variation (spectral variability).

To quantify acoustic dissimilarities, Representational Dissimilarity Matrices (RDMs) were computed using correlation distance (1−Pearson’s r). One RDM was based on vectorized average MPS, and another on a set of acoustic features (PSSD, PSCV, Root Mean Square (RMS) amplitude, fundamental frequency (F0) mean and standard deviation, and HNR; Table 1). Hierarchical clustering applied to these RDMs generated dendrograms (Fig. 2C: left, MPS-based; right, acoustic feature-based) to visualize the grouping structure. Both dendrograms effectively represented their respective data, as indicated by high cophenetic correlation coefficients (r = 0.94 for MPS-based, r = 0.80 for feature-based). However, the RDMs themselves were only weakly correlated (Spearman’s ρ = 0.41), suggesting they captured distinct acoustic properties. Both dissimilarity analyses grouped nature sounds with animal calls. Based on the MPS analyses, coos were the most distinct category, while based on other acoustic features, aggressive calls were intermediate between non-vocalizations and the other two vocalization types.

A scrambled version of each of the five categories was generated using a *time-domain scrambling* method (Ellis, 2025). We divided the waveforms into multiple frequency bands using gammatone filters, which are a linear approximation of the frequency resolution of the ear. Each band was filtered twice, once forwards and once backwards in time, by segmenting them into short time windows, applying a raised cosine taper, shuffling the segments, and then re-overlapping them (Fig. 2E).

Spectral centroids (amplitude-weighted mean frequencies; Fig. 2D) significantly differed across the ten sound categories, including both natural and scrambled versions (Friedman test: χ²(9) = 57.03, p = 4.98 × 10^-9^; N = 10 stimuli per category). Post-hoc Bonferroni-corrected comparisons revealed that screams had a significantly higher spectral centroid than coos (p < 0.05), scrambled coos (p < 0.001), nature sounds (p < 0.001), scrambled nature sounds (p < 0.001), animal calls (p < 0.05), and scrambled animal calls (p < 0.05). Scrambled screams also showed significantly higher centroids than scrambled coos (p < 0.001), nature sounds (p < 0.001), and scrambled nature sounds (p < 0.001). These results suggest that high-frequency energy is a distinguishing feature of screams and their scrambled versions relative to most other categories. Screams also had the highest average fundamental frequency (F0; 684 Hz). Non-vocalizations exhibited lower and more variable F0s (e.g., animal calls: 503 Hz; nature sounds: 440 Hz). Moreover, F0 standard deviation—indicative of pitch modulation—was larger in aggressive calls and screams, whereas coos displayed very stable pitch contours (F0 SD: 54.13 Hz; Table 1).

Despite clear distinctions, some acoustic features showed partial overlap across categories. Aggressive calls resembled non-vocalizations in spectral spread but aligned with screams in terms of pitch modulation and variability. Animal calls shared similar HNR with screams, suggesting converging harmonic properties. Coos stood out as the most distinct category in modulation patterns (MPS), though less so in other features, highlighting the multidimensional nature of acoustic structure. Overall, the stimulus set exhibited both categorical diversity and graded acoustic relationships, which likely determines perception and neural responses.

### 2.5 Scanning procedures

All acquisitions were performed with a 3T Siemens PrismaFit scanner. A 16 (M58) or 10 (M50) channel phased array receive coil was embedded in the headpost of each monkey, just above the skull in order to improve the sensitivity of the sub-millimeter resolution fMRI measurements (Janssens et al., 2012). To further increase the signal to noise ratio (SNR), a contrast agent, composed of monocrystalline iron oxide nanoparticles, Molday ION^TM^, was injected into the animal’s saphenous vein (9–11 mg/kg) prior to each functional scanning session (Leite et al., 2002; Vanduffel et al., 2001). The injections were administered while the monkeys were awake as they had been trained to remain calm during the procedure.

#### 2.5.1 Anatomy

Before we started the fMRI recording sessions, a high-resolution (0.4 mm isotropic voxel size) T1-weighted scanning session was performed, while the subjects were anesthetized with ketamine-xylazine (M50) or ketamine-medetomidine (M58). For this purpose, we used a custom-built local single-loop receive coil and the body transmit coil of the scanner. We acquired 13 and 11 T1-weighted 3D images for subjects M50 and M58 using a 3D MPRAGE sequence (TR = 2700 ms; TE = 3.5 ms; in-plane FOV 104 × 128 mm; matrix size 250 × 320; slices = 208; = 9°; TI = 882 ms). For M50 and M58, we obtained 5 and 4 T2-weighted 3D images, respectively, using the same in-plane FOV and matrix size as the T1-weighted images. This approached aimed to reduce field inhomogeneities in the anatomical images and to extract dura and blood vessels from pial surfaces (Glasser et al., 2013), using a sampling perfection with variable flip angle turbo spin-echo sequence (SPACE) sequence (TR = 3200 ms; TE = 456 ms; total turbo factor = 131; echo spacing = 6 ms; no fat suppression). We adapted the non-human primate version of the Human Connectome Project (HCP) pipeline (Autio et al., 2020).

#### 2.5.2 fMRI

fMRI images were acquired from awake macaque monkeys using 16-channel (M58) and 10-channel (M50) implanted receive coils, along with a custom-built local single loop transmit coil. We acquired gradient-echo echo-planar images (GE-EPI) with 0.6 mm isotropic voxels, covering the entire brain (Repetition time (TR) = 3000 ms; echo time (TE) = 21 ms; accelerated multiband (MB) = 2; acceleration factor = 3; in-plane field of view (FOV) 84 × 84 mm; matrix size 140 × 140; the flip angle = 90°). The number of slices was adjusted according to individual brain size, resulting in 90 and 80 slices in M58 and M50, respectively.

Each run lasted for 243 pulses, and 44 runs were acquired for M58 and 46 for M50, and fixation performance exceeded 95% within the eye fixation tolerance window of 2 degrees.

Data were pre-processed and analyzed using a combination of FSL (Jenkinson et al., 2012), SPM software (version SPM12, Wellcome Department of Cognitive Neurology, London, UK, https://www.fil.ion.ucl.ac.uk/spm/software/), JIP analysis toolkit (http://www.nitrc.org/projects/jip), Advanced Normalization Tools (ANTs), and workbench (https://www.humanconnectome.org/software/get-connectome-workbench).

After stripping the skull from the epi volumes, a template was created from the first 10 volumes of the first acquisition day. Additionally, we applied a fieldmap correction and motion correction using 6 degrees of freedom. To reduce frame-to-frame distortions, a slice-by-slice distortion correction was implemented using a B-spline grid-based nonlinear registration method (Kolster et al., 2009), aligned to the same template. Spatial smoothing was applied with a Gaussian Kernel of 1-mm full width of half maximum.

Subsequently, a generalized linear model (GLM) denoising algorithm (Kay et al., 2013) was applied to filter out physiological and other nuisance temporal noise, resulting in enhanced signal-to-noise ratios. After converting the time series into percent signal change, this technique identifies noise voxels that do not correlate with the task, specifically vertices with negative R^2^ values in the task-based model. Noise regressors were derived from the noise time series using principal components analysis, which were then progressively removed from the time series of all vertices, one at a time. The optimal number of noise regressors was determined by assessing the cross-validated R^2^ improvement observed in the task-based model. Finally, the denoised images were obtained by removing the nuisance components.

The denoised and smoothed data were utilized for the GLM and percent signal change analysis but not for the MVPC analysis, to preserve the fine spatial resolution of acquired images.

Various analyses were performed in the functional space of the monkeys and the results were then coregistered, using linear and non-linear registration, to their high-resolution T1 using the antsRegistration command line. The high-resolution T1-weighted images were processed following to the HCP pipeline (Autio et al., 2020; Glasser et al., 2013).

Functional images were also normalized into the MEBRAINS template (Balan et al., 2024) to perform analysis with the two monkeys combined in the same space.

### 2.6 Analysis

#### 2.6.1 General linear model (GLM) analysis

SPM (version SPM12, Wellcome Department of Cognitive Neurology, London, UK, https://www.fil.ion.ucl.ac.uk/spm/software/) was used to perform a General Linear Model (GLM) analysis on the preprocessed data. These analyses estimated model parameters (beta coefficients) for task-related regressors, from which statistical parametric maps (e.g., t-maps) were generated for specific contrasts of interest. For each run, five dummy scan images were acquired at the beginning to reach stability. These were not included in the statistical analyses. Head motion parameters, derived from the realignment step, were included in the GLM as regressors of no interest to account for movement-related variance. The modeled neural responses were convolved with an MION-specific hemodynamic response function (HRF) as described by Vanduffel et al. (2001).

Fixed-effects analyses were conducted for each monkey individually. Statistical significance was assessed using a voxel-level threshold of p < 0.05, corrected for multiple comparisons across the whole-brain volume using the family-wise error rate (FWE), which corresponded to a t-score threshold of approximately 5.1. Additionally, results were explored at an uncorrected voxel-level threshold of p < 0.001 (t-score 3.09), without any cluster-extent correction applied.

Another fixed-effect analysis was performed on the combined data from the monkeys normalized to the MEBRAINS common template. Statistical significance was assessed using a primary voxel-level threshold of p < 0.05 (FWE corrected, t-score: 5.1) and uncorrected voxel-level threshold of p < 0.001 (t-score 3.09). This time, a cluster-level threshold of p < 0.05, corrected for multiple comparisons using the False Discovery Rate (FDR), with a minimum cluster extent of 10 contiguous voxels was applied (p < 0.05 FDR (cluster-level correction) if kE ≥ 10 voxels).

#### 2.6.2 ROI analysis

Potential regions-of-interest (ROIs) for detailed analysis were identified as significant activation clusters that met stringent statistical criteria for the “vocalizations > non-vocalizations” contrast. This process involved a two-step thresholding procedure:

1) Based on the combined data from both monkeys, activation clusters had to meet statistical significance at a voxel-wise threshold of p < 0.05 Family-Wise Error (FWE)-corrected, and pass a 10 voxels cluster-level threshold of p < 0.05 False Discovery Rate (FDR)-corrected (MEBRAINS template space).
2) Furthermore, areas within these clusters had to show statistically significant activation at an uncorrected threshold of p < 0.001 (without cluster correction) in response to vocalizations versus non-vocalizations in each monkey and each hemisphere separately (individual monkey space). This individual-level thresholding was performed on half of the individual data for each monkey, that is, 22 runs for M58 and 23 runs for M50 (independent from the other half used for subsequent ROI analyses, Fig. 1C). The activity profiles of each ROI were then extracted on the other half of the data using the MarsBar SPM toolbox (Brett et al., 2002); marsbar.sourceforge.net). The mean percent signal change (+/- standard error of the mean across runs; % signal change) was calculated for each condition relative to the silent baseline. Percent signal change was compared using Friedman non-parametric tests and Wilcoxon nonparametric paired tests. Finite Impulse Response (FIR) modelling was also used to estimate the event-related activity from stimulus onset to the end of the baseline block (Supplementary Fig. S2). For each condition within each ROI, the mean FIR time course and the corresponding standard error of the mean (SEM) across trials were calculated. These time courses were then smoothed using a 3-point moving average for visualization purposes. Supplementary Figure S2 also shows, for each ROI, the time course of percent signal change (mean ± SEM) across a run. ROI time courses were extracted from runs sharing the same block organization. Percent signal change was first computed relative to the mean of the silent block, and then averaged across runs at each time point. For visualization purposes, the resulting group-level time course was baseline-corrected by subtracting the signal at the beginning of the run.

### 2.7 MVPA

To examine the encoding of specific vocalizations in brain areas, we conducted a *multivariate pattern classification analysis* (MVPC; see fig. 1D). This analysis utilized the *support vector machine* (SVM) method implemented in the CoSMoMVPA toolbox (Oosterhof et al., 2016) within MATLAB. For each monkey and each region of interest (ROI) of each hemisphere, an SVM classifier was trained on the β-patterns associated to each sound category from (NRuns – 1) runs, and then tested on data from the held-out run, resulting in a 22-fold cross-validation for M50 and a 23-fold cross-validation for M58. Classification accuracy was calculated for each fold and averaged across folds to provide a mean accuracy.

For the classification of three vocalizations versus three scrambled vocalizations categories, a pairwise method was applied. This approach enables us to understand how the brain distinguishes between various types of vocalizations and their scrambled counterparts, offering insights into the neural representation of complex acoustic structures. To assess the statistical significance of individual classification performances, a one-tailed Wilcoxon rank-sum test was performed, comparing accuracies per fold to chance level (1/2 for 2-class, 1/3 for 3-class). To examine the statistical differences in accuracy across classifiers with different combinations, a Kruskal-Wallis test was performed. This comprehensive analysis allows us to understand the brain’s ability to distinguish between different vocalization types and their scrambled counterparts, hence, providing insights into the neural representation of complex acoustic structures.

#### 2.7.1 Representational geometry

Additionally, a dissimilarity matrix (*DSM*) was computed to visualize the representational geometry of different sound categories within each ROI. This analysis further clarifies the relationships and distinctions among neural responses to various sounds. For each ROI, β-patterns for each sound category were first averaged across runs. To prevent spuriously high correlations, we subtracted the average value across all categories before calculating the dissimilarities. Next, we computed Euclidean distances between every pair of response patterns. The dissimilarity matrix (DSM) was then constructed from the condition-by-condition dissimilarity. Finally, to visualize the DSM for each ROI, we performed non-classical multi-dimensional scaling on the matrix and projected the dissimilarity relationship onto a two-dimensional space.

#### 2.7.2 Multiple regression RSA (representational similarity analysis)

To evaluate the contributions of representational models to the dissimilarity structure in each ROI, we conducted a multiple linear regression RSA on the neural dissimilarity matrix. Specifically, we defined two types of target models. The first, the vocalizations versus non-vocalizations dissociation model, posits maximum similarity (0) among the three types of vocalizations and two types of non-vocalizations, with maximum dissimilarity (2) between vocalizations and non-vocalizations. The second model, the Vocalization distinction model, assumes maximum dissimilarity among vocalizations (2), maximum similarity among non-vocalizations (0), and intermediate similarity (1) between vocalizations and non-vocalizations.

Before conducting the regression, both the neural DSM and the model DSMs were normalized using z-score transformation. The neural DSM was then regressed on the model DSMs to estimate regression coefficients (β) for each model. To estimate the distribution of β values, we employed a repeated hold-out method, where we randomly held out (NRuns – 3) runs to compute the DSM for regression, repeating this process 1000 times to report the average β values. To assess the significance of coefficients, we conducted the Wilcoxon signed-rank test against zero.

By employing these different types of MVPA, we obtain complementary insights in vocalization processing: the 3-class classification offers a detailed understanding of how specific types of vocalizations are processed and how they differentiated from their scrambled versions. Meanwhile, the DSM offers a visual representation of the similarity relationships, enhancing our comprehension of the underlying neural encoding mechanisms across different sound categories.

## 3 Results

Two monkeys, M58 and M50, equipped with implanted 16 and 10-channel receive coils respectively, were exposed to a variety of sounds: coos, screams, aggressive calls, nature sounds, calls from non-conspecific animals, scrambled versions of these calls, in a randomized blocked fMRI design. Silent blocks alternated with each sound block (Fig. 1B). We first discuss the overall brain activations in response to the vocalizations, followed by an analysis of the selectivity for different types of vocalizations.

### 3.1 Whole-brain activations to monkey vocalizations

**Fig. 3.**
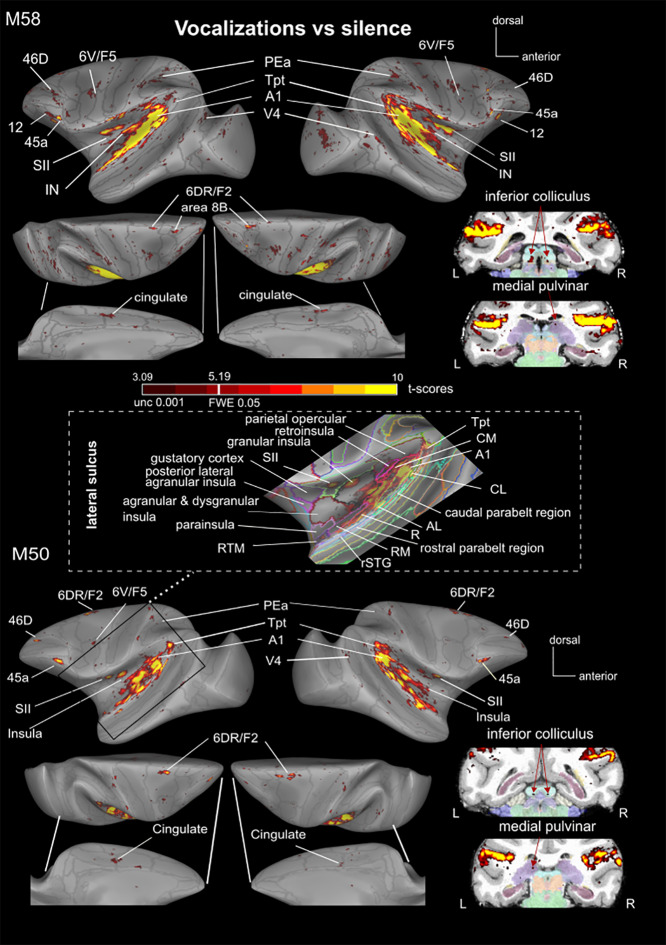
Main auditory (sub)cortical activations in both monkeys. Projection of SPM *t*-score maps (p < 0.001 uncorrected, t > 3.09; p < 0.05 FWE corrected, t-score> 5.1, fixed effect—see color scale bar) onto surface representations of the left and right hemispheres for M58 and M50, comparing “vocalizations” to “silence”. The *t*-score map highlighting main auditory activations is also overlaid on coronal sections at the level of the inferior colliculus and the medial pulvinar. Gray lines on the surface delineate the CHARM atlas (Jung et al., 2020; Reveley et al., 2017), while the SARM atlas (Hartig et al., 2021) superimposed on the coronal section to indicate different subcortical regions. The middle inset represents an enlarged view of the lateral sulcus with the activations of M50’s left hemisphere overlying the anatomical regions of the CHARM atlas as indicated with different colors.

We first compared brain activity evoked by conspecific vocalizations—comprising coos, aggressive calls, and screams—against silence. This analysis revealed a widespread network of auditory activity in both monkeys, covering much of the posterior bank of the lateral sulcus but also some areas in its fundus and its anterior bank (p < 0.05, corrected FWE). Key regions included primary auditory cortex (A1), as well as the higher-level auditory areas such as the caudomedial area (CM), caudolateral area (CL), anterolateral area (AL), rostral area (R), rostromedial area (RM), and lateral rostrotemporal area (RTL) of the auditory cortex (Fig. 3; see for detail Supplementary Tables S1 and S2).

In addition to auditory regions, vocalizations also elicited fMRI responses in visual areas V1 and V4, the rostral superior temporal gyrus (rSTG), the temporo-parieto-occipital region (TPO), parietal areas PEa and VIP, and regions within the insula (granular insula, Ig, dysgranular insula, Id and retroinsula Ri), in both monkeys and in both hemispheres (thresholds: p < 0.05, corrected FWE and p < 0.001 uncorrected). The primary and secondary somatosensory cortex (S2), cingulate cortex (area 24c’), and dorsal premotor cortex, including 6DR (also known as F2) and premotor area 6 V, were also activated by vocalizations, along with areas 45 and 46D in prefrontal cortex. In M50, activations in both 6DR and 6 V were significant at the corrected threshold. In M58, only left 6DR survived the corrected threshold, whereas right 6DR and bilateral 6 V were only significant at the uncorrected threshold, suggesting a difference in the strength of activation between these premotor subregions. In M58, activations were observed in area 8B, as well as in areas 12 m and 13 l of the orbitofrontal cortex. Combining the two monkeys in the same space to perform a single analysis also revealed area 8B and orbitofrontal area 12 l, in addition to the regions already described above (Fig. 5A and Supplementary Table S3). At the subcortical level, the bilateral inferior colliculus and the right medial pulvinar were activated at uncorrected level (p < 0.001). Additional consistent bilateral activations were observed at the uncorrected threshold (p < 0.005) in both monkeys, particularly in the claustrum and medial geniculate nucleus (see Supplementary Tables S1 and S2). When we performed a group analysis and spatially smoothed the data at 3 mm, the activations in the MGN and IC became detectable at a threshold of p < 0.05 (FWE corrected) and p < 0.001 (uncorrected level), respectively. This was not the case for the pulvinar and claustrum, which may indicate inter-subject variability of the exact spatial locations of activations induced by vocalizations (versus silence) in these regions.

**Fig. 4.**
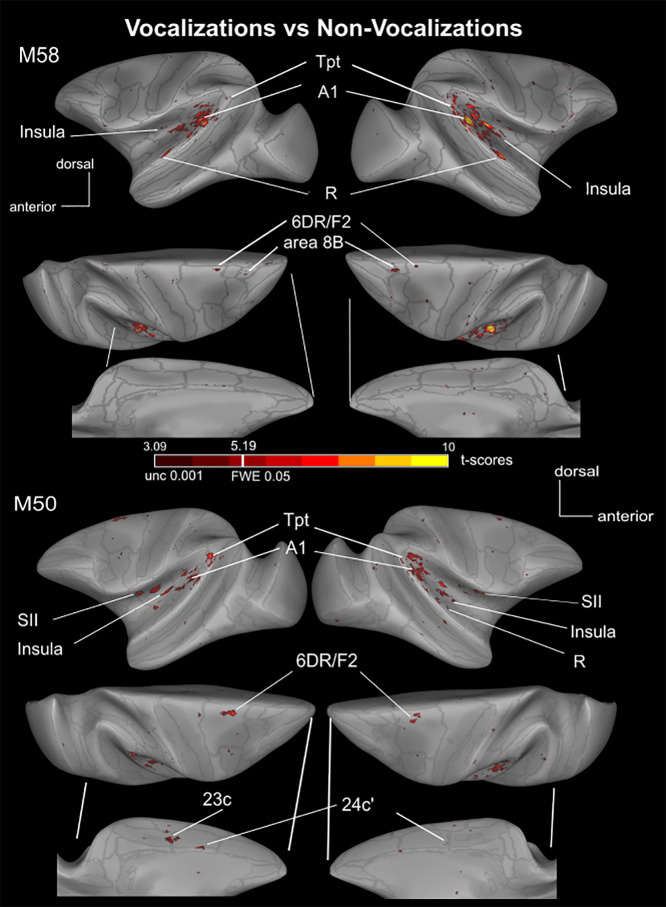
Main vocalization-specific cortical activations in both monkeys. The SPM *t*-score map (p < 0.001 unc, t-scores> 3.09; p < 0.05 FWE corrected, t-score> 5.1, fixed effect; see color code scale) is projected onto surface representations of the left and right hemispheres of M58 and M50, for the contrast “vocalizations” versus “non-vocalizations”. Gray lines on the surface represent the delineation of regions according to the CHARM atlas.

**Fig. 5.**
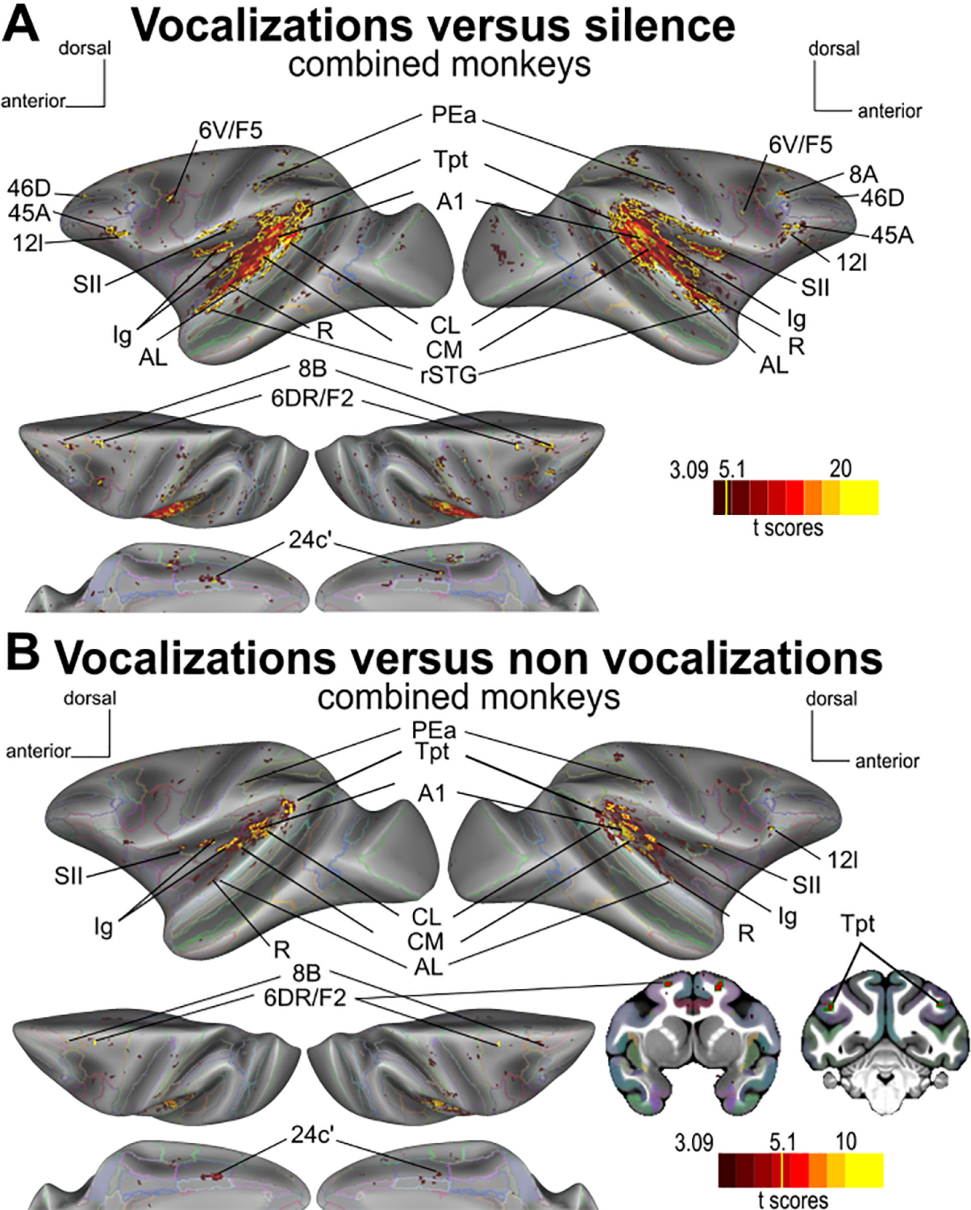
Main auditory cortical activations for combined monkeys (M58 and M50). (A) Projection of SPM t-score maps (p < 0.001 uncorrected, t > 3.09; p < 0.05 FWE corrected, t-score> 5.1 outlined in yellow, fixed effect—see color scale bar) onto surface representations of the left and right hemispheres of the MEBRAINS template (Balan et al., 2024), comparing “vocalizations” to “silence” (A) and “vocalizations” to “non-vocalizations” (B). Coronal sections are presented in B at the level of areas 6DR/F2 and the Tpt, respectively. With t-score> 5.1 outlined in green. Colored lines on the surface delineate the CHARM atlas (Jung et al., 2020; Reveley et al., 2017). SII: secondary somatosensory cortex; Ig: granular insula; Id: dysgranular insula; Ri: retroinsula; CM: caudomedial belt region; A1: primary auditory cortex; Tpt: temporoparietal area; CL: caudolateral belt region; AL: anterolateral belt region; R: rostral core region. rSTG: rostral superior temporal sulcus. The activations are described in Supplementary Table S3.

When comparing vocalizations to non-vocalizations (the latter consisting of animal calls and nature sounds), several auditory regions were more active in response to conspecific vocalizations (Figs. 4 and 5B). These vocalization-specific regions, found in both monkeys and hemispheres, included the caudal part of the lateral sulcus (Tpt), CL, A1, AL, and an activation spot within the dorsal premotor area 6DR/F2. Some regions were selectively activated in only one of the two monkeys, such as the secondary somatosensory cortex (SII), the granular insula (Ig), area 24c’, MST, and area 6 V in M50, and areas F1 and 8B in M58. The most rostral activation specific to vocalizations in the lateral sulcus was the rostral core region (R). However, this region was only identifiable at an uncorrected level (p < 0.005) in the left hemisphere of M50 (t-score = 3.097). Therefore, it is not apparent in Figure 4. Details of these vocalization-specific activations are presented in Supplementary Tables S1 and S2. Combining the two monkeys, specific activations for vocalizations versus non-vocalizations with an FWE corrected threshold of 0.05 appear to be present in areas 6DR/F2, SII, Ig, A1, CL, Pea, and Tpt in both hemispheres (in bold in Table 2 last column). Area 8B, CL, CM, AL, and R are more activated by vocalizations than non-vocalizations, but at uncorrected level or only in one hemisphere with the 0.05 FWE threshold (p < 0.001, Figure 5; see Table 2 and Supplementary Table S3 for more details). Note that all these clusters pass the 10-voxel cluster 0.05 FDR corrected threshold except area 8B.

**Table 2.** vocalizations > silence, and vocalizations > non-vocalizations maxima of combined monkeys (MEBRAINS space).

Combined monkeys (MEBRAINS space)					
Label D99 (CHARM 6)	Peak coordinates (mm)			T scores vocalizations vs silence	T scores vocalizations vs non-vocalizations (voxel counts per cluster (kE))
L 8B	-19	19	61	**8.6**	4.7 (4 vox)
R 8B	18	17	64	**15**	3.96 (22 vox)
**L 6DR/F2**	-19	-4	65	**9.3**	**7.7 (24 vox)**
**R 6DR/F2**	20	-3.6	63	**9.1**	**6.1 (16 vox)**
L SII	-50	-10	28	**9.5**	**6.08 (34 vox)**
R SII	53	-8	28	**10.65**	**7.9 (50 vox)**
L Ig	-41	-36	26	**11.02**	**6.8 (20 vox)**
R Ig	40	-38	27	**16.18**	**8.3 (23 vox)**
L CM	-44	-48	30	**27.8**	**8.7 (24 vox)**
R CM	37	-47	32	**6.04**	**5.3 (41 vox)**
**L A1**	-47	-48	34	**54**	**8.6 (97 vox)**
**R A1**	47	-50	32	**47.7**	**10.7 (139 vox)**
**L Tpt**	-47	-65	47	**10.2**	**6.65 (48 vox)**
**R Tpt**	41	-61	45	**7.8**	**5.3 (44 vox)**
L CL	-56	-50	32	**26.6**	**6.83** (11 vox)
R CL	59	-49	33	**23.7**	4.9 (18 vox)
L AL	-70	-23	22	**47.4**	4.9 (20 vox)
R AL	63	-29	22	**57.2**	**7.2 (10 vox)**
L R	-59	-26	15	**16.8**	3.8 (25 vox)
R R	58	-27	18	**14.8**	4.6 (10vox)
L PEa (5)	-53	-32	52	**8.3**	**5.3** (16 vox)
R PEa (5)	50	-39	49	**7.2**	**5.1 (35 vox)**

Related to Figure 5. bold: p < 0.05 FWE corrected, t-score> 5.1. p < 0.05 FDR corrected, t-score> 4.43; p < 0.001 uncorrected, t-scores> 3.09; p < 0.005 uncorrected, t-score >2.58. In bold the label of region selected for the ROI analysis.

SII: secondary somatosensory cortex; Ig: granular insula; CM: caudomedial belt region; A1: primary auditory cortex; Tpt: temporoparietal area; CL: caudolateral belt region; AL: anterior lateral belt; R: rostral core region.

### 3.2 Areas Tpt and 6DR selectively discriminate vocalization types

Significant activation clusters for the “vocalizations > non-vocalizations” contrast were identified using a stringent two-step statistical thresholding procedure to define the regions-of-interest (ROIs) for the next analysis. An area was considered for ROI selection if it met the following criteria: (1) In the combined data from the two monkeys (Fig. 5B and Table 2 and Supplementary Table S3), it showed statistically significant activation at a voxel-wise threshold of p < 0.05 Family-Wise Error (FWE)-corrected, and the activation cluster containing these voxels passed a cluster-level threshold of p < 0.05 False Discovery Rate (FDR)-corrected with a minimum size of 10 contiguous voxels. (2) Furthermore, to ensure robust activation in individual subjects for ROI analysis in individual space (which offers better spatial precision and reproducibility, yet less statistical power), areas within these clusters had to show statistically significant activation at an uncorrected threshold of p < 0.001 (without cluster correction) in response to vocalizations versus non-vocalizations in each monkey and each hemisphere separately. This individual-level thresholding was performed on half of the individual data for each monkey, independent from the other half used for subsequent ROI analyses (such as percent signal change calculation, time course analysis, MVPA analysis, see Fig. 1C).

**Fig. 6.**
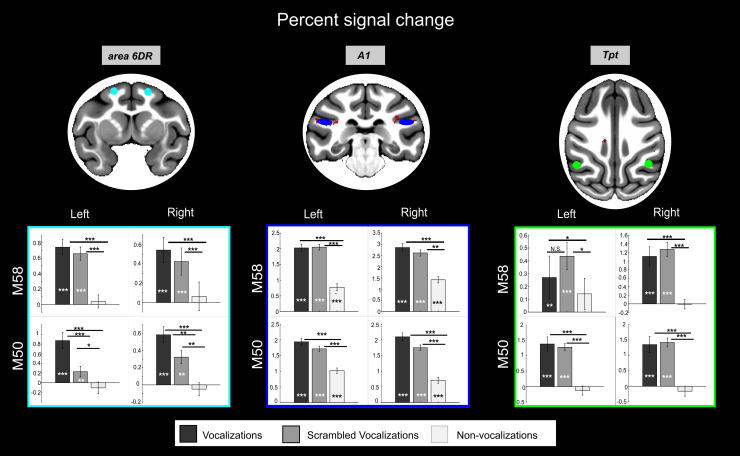
Percent signal change analysis for both monkeys. For each region of interest (ROIs), the percent signal change is shown for vocalizations independent of vocalization type (black), scrambled vocalizations (dark gray), and non-vocalizations (light gray). Comparisons between conditions were performed using the Wilcoxon nonparametric test (***p < 0.001; **p < 0.01; *p < 0.05). In this figure, ROIs are displayed in the MEBRAINS template. ROIs are displayed on both the surface and 3D brain volume in individual subject space in Supplementary Figure S1. The color of the boxes with plots corresponds to the ROIs color.

**Fig. 7.**
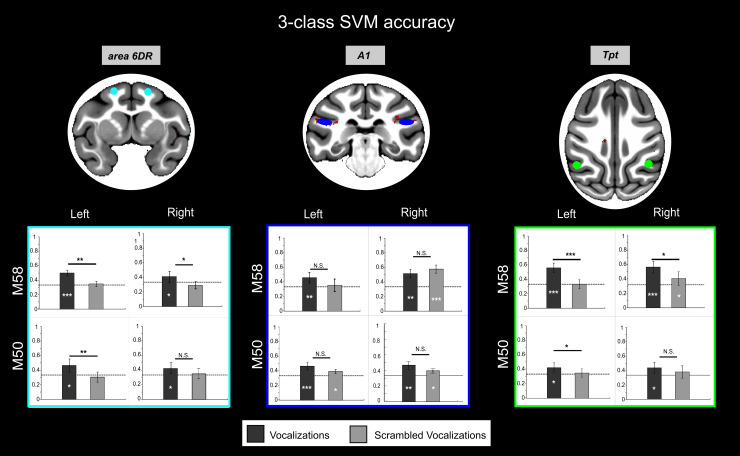
3-class classification multivariate pattern classification analysis. For each region of interest (ROIs), the accuracies of the 3-class SVM classification for vocalizations type (black) and scrambled vocalizations type (light gray) are also shown. Comparisons between conditions were performed using the Wilcoxon nonparametric test (***p < 0.001; **p < 0.01; *p < 0.05). In this figure, ROIs are displayed in the MEBRAINS template. ROIs are displayed on both the surface and 3D brain volume in individual subject space in Supplementary Figure S1. The color of the boxes with plots corresponds to the ROIs color.

Two regions hypothesized to be key nodes in primate auditory processing streams, potentially being involved in processing complex vocalizations and linking auditory input to motor systems, fulfilled the criteria for further analyses: area 6DR/F2 and area Tpt. Dorsal premotor area 6DR/F2 is likely a key component of the putative dorsal auditory-motor pathway, given its anatomical connectivity to temporal regions. Moreover, emerging comparative evidence also suggests its involvement in vocal perception, even without overt production (Dureux et al., 2024; Jafari et al., 2023). Area Tpt is also implicated due to its established anatomical connections with frontal areas via the arcuate fasciculus and previous suggestions regarding its role in higher-level auditory processing, including vocalizations (Gil-da-Costa et al., 2006; Romanski & Averbeck, 2009). Within the lateral sulcus areas responding to both criteria, we chose to select area A1 as control, as this primary auditory region is generally responsive to sounds. Figures 6 and 7 respectively show the percent signal changes and 3-class multi voxel pattern analysis (MVPA) for 6DR (cyan), Tpt (green) and A1 (blue) for monkeys M50 and M58 (ROIs are displayed on both the surface and 3D brain volume in individual subject space in Supplementary Fig. S1). Both these analyses were performed on the second half of the data. Additionally, Supplementary Figure S2 shows for each of these three ROIs the time courses across an entire run, as well as the average MION signal modulation from stimulus onset to the end of the baseline block.

The control ROI corresponding to A1 showed significant activation comparing the three stimulus types (vocalizations, scrambled vocalizations, and non-vocalizations; Wilcoxon nonparametric test p < 0.001, Fig. 6) to silence. There was no average activity difference between vocalizations and their scrambled version, but both induced higher responses than non-vocalizations (Wilcoxon nonparametric test p < 0.001, Fig. 6). A1 also demonstrated above-chance categorical discrimination accuracy for both the vocalizations and scrambled vocalizations (Fig. 7). However, there was no significant difference in decoding accuracies between vocalizations and non-vocalizations in either hemisphere of both monkeys.

The ROIs in area 6DR showed significant differences in percent signal changes when comparing vocalizations (p < 0.001) and scrambled vocalizations (p < 0.001 M58, p < 0.01 M50) to silence, but not when comparing non-vocalizations to silence (Fig. 6). Moreover, the average activity in these ROIs was higher for vocalizations compared to non-vocalizations for both monkeys and to both scrambled vocalizations for M50. Our 3-class MVPA analyses for area 6DR revealed above-chance discrimination accuracies between vocalization types (Wilcoxon nonparametric test p < 0.001 M58, left hemisphere; p < 0.05; M50, left and right hemispheres), except for the right hemisphere in M58 (Fig. 7). Furthermore, this accuracy was higher than the discrimination accuracy for scrambled vocalizations (Wilcoxon nonparametric test p < 0.01 in the left hemisphere of both monkeys; p < 0.05 in right hemisphere of M58; N.S. in right hemisphere of M50).

The ROI located caudally in the lateral sulcus corresponds anatomically to the Tpt region in the D99 and CHARM atlases. The fMRI pattern of activity measured in Tpt is comparable to that observed in area 6DR/F2, as it responds to vocalizations (p < 0.001 for M50; p < 0.01 for M58 in the left hemisphere) and scrambled vocalizations (p < 0.001), but not to non-vocalizations (relative to silence) (Fig. 6). Furthermore, the pattern of activity in Tpt can discriminate vocalization types above chance (p < 0.001 in both hemispheres of M58, and the left hemisphere of M50; p < 0.05 in the right hemisphere of M50) but in most cases not scrambled vocalizations (only p < 0.05 in the right hemisphere of M58). The discrimination accuracy was, thus, higher for vocalizations than scrambled vocalizations (p < 0.05 M50 left hemisphere; N.S. right hemisphere; M58 p < 0.001 left hemisphere and p < 0.05 right hemisphere) (Fig. 7).

### 3.3 Representational similarity analyses (RSA) confirm selective vocalization coding in areas Tpt and 6DR

**Fig. 8.**
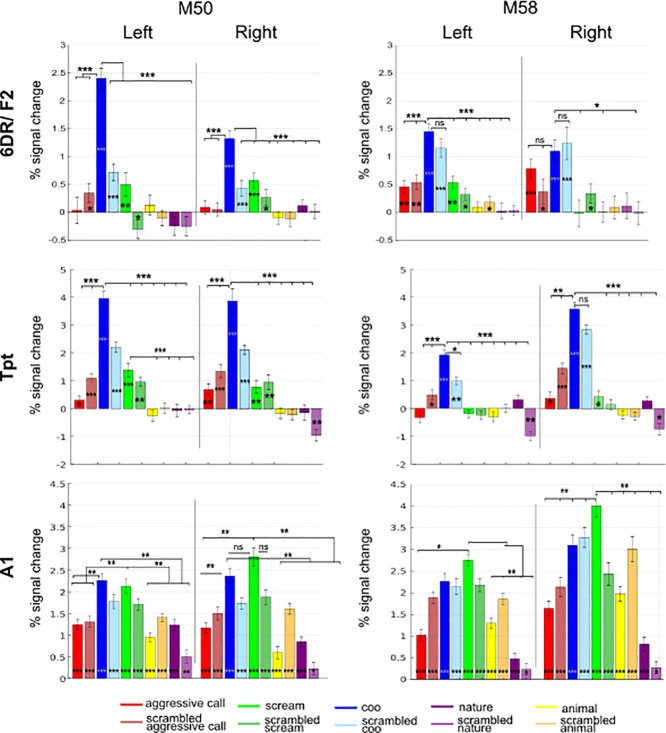
Percent signal change for each condition compared to silent blocks per hemisphere and subject. The conditions are post-hoc compared with each other via Wilcoxon signed-rank test with Bonferroni correction (stars at the top of the bar plot). They are also compared to silence with the same test (stars at the bottom of each bar) (***p < 0.001; **p < 0.01; *p < 0.05).

To better characterize the selectivity of the extracted ROIs for different types of vocalizations, we compared the percent signal changes evoked by all stimuli within the previously selected regions (see Fig. 8). Again, we used independent data for ROI selection and analyses. All ROIs demonstrated a significant difference between conditions (Friedman test: L A1: *M58* p = 1.5 x 10^-21^, χ²(9) = 119.74, *M50* p = 5.7 x 10^-22^, χ²(9) = 121.78; R A1: *M58* p = 5.4 x 10^-26^, χ²(9) = 141.33, *M50* p = 1.04 x 10^-26^, χ²(9) = 144.8; L R: *M58* p = 7.6 x 10^-12^, χ²(9) = 71.52, M50 p = 2.06 x 10^-7^, χ²(9) = 48.49; R R: *M58* p = 8.2 x 10^-16^, χ²(9) = 91.47, M50 p = 3.7 x 10^-14^, χ²(9) = 104.16; L Tpt: *M58* p = 2.6 x 10^-16^, χ²(9) = 93.86, M50 p = 3.6 x 10^-22^, χ²(9) = 122.73; R Tpt: *M58* p = 2.4 x 10^-21^, χ²(9) = 118.8, M50; L 6DR: *M58* = 2.5 x 10^-14^, χ²(9) = 84.01, M50 p = 5.2 x 10^-13^, χ²(9) = 77.42; R 6DR: *M58* p = 2.1 x 10^-5^, χ²(9) = 37.53, *M50* p = 2.4 x 10^-9^, χ²(9) = 28.60)*.* Therefore*,* we performed post hoc comparisons, using a Wilcoxon signed-rank test with Bonferroni correction, to identify specific differences in activity between pairs of conditions (p values are summarized in Supplementary Tables S3, S4, S5, and S6).

In area 6DR, coos elicit more fMRI activity than most other conditions, which probably causes the observed differences between the vocalizations and non-vocalizations discussed in the previous section (upper row of Fig. 6; M50: coos higher than all the conditions p > 0.001 both hemispheres; M58: coos higher than all other conditions at p > 0.001, except for the scrambled coos in the left hemisphere, coos higher than screams, animal calls and nature sounds at p > 0.05 in the right hemisphere; see Supplementary Table S4 for the statistics). Possibly, the scrambled versions of coos may still be perceived as coos by the subjects. Importantly, unlike aggressive calls and screams, neither the non-vocalizations nor their scrambled versions activated this area, except for the left hemisphere of M58 which shows activation for scrambled animal calls. The activation pattern in Tpt resembles that of 6DR (see Supplementary Table S5 for detailed statistics), showing significant responses to vocalizations and their scrambled versions, with a clear preference for coos and no activation to animal or nature sounds.

Unlike 6DR and Tpt, area A1 is strongly activated by all sounds (except the scrambled nature sounds). It exhibits higher activation for the screams compared to most other categories in the right hemisphere of both monkeys and the left hemisphere of M58, as well as a preference for coos versus aggressive calls (see Supplementary Table S6 for the p values).

The variation in activity based on vocalization type does not follow a uniform pattern across the different ROIs. Areas Tpt and 6DR displayed a preference for affiliative vocalizations (coos) compared to screams or aggressive calls, whereas area A1 and R (in M58) showed higher activity for screams. These differential preferences across regions appear to be related to the distinct acoustic characteristics of the sound categories. Based on our acoustic analysis (Table 1, Fig. 2), screams are characterized by broadband, noisy spectro-temporal energy (high spectral centroid, relatively low HNR, high F0 variability) and appear acoustically distinct from other categories (Fig. 2C). When we analyzed the frequency weighted by the amplitude of the sounds, screams and their scrambled version were distinct from the other sound types (Fig. 1B). The stronger activity for screams observed in primary auditory cortex (A1) and area R (in M58) is consistent with the known role of early auditory areas in processing these types of salient, broad spectral features and rapid acoustic changes.

In contrast, coos exhibit a more tonal, harmonic structure (relatively high HNR, lower F0 variability, specific mean F0 range) with distinct spectro-temporal patterns (Table 1, Fig. 2). The preferential response to coos in areas Tpt and 6DR suggests a tuning in these regions to the specific acoustic regularities or harmonic content present in coos, potentially reflecting their importance as affiliative social signals. This explanation does not appear to be solely accounted for by simple low-level properties common across categories, as indicated by the differing patterns in A1. Possibly, monkeys are more reactive to these distinct and socially relevant acoustic features of affiliative coos than the other vocalizations tested. Further investigation into this phenomenon is warranted.

**Fig. 9.**
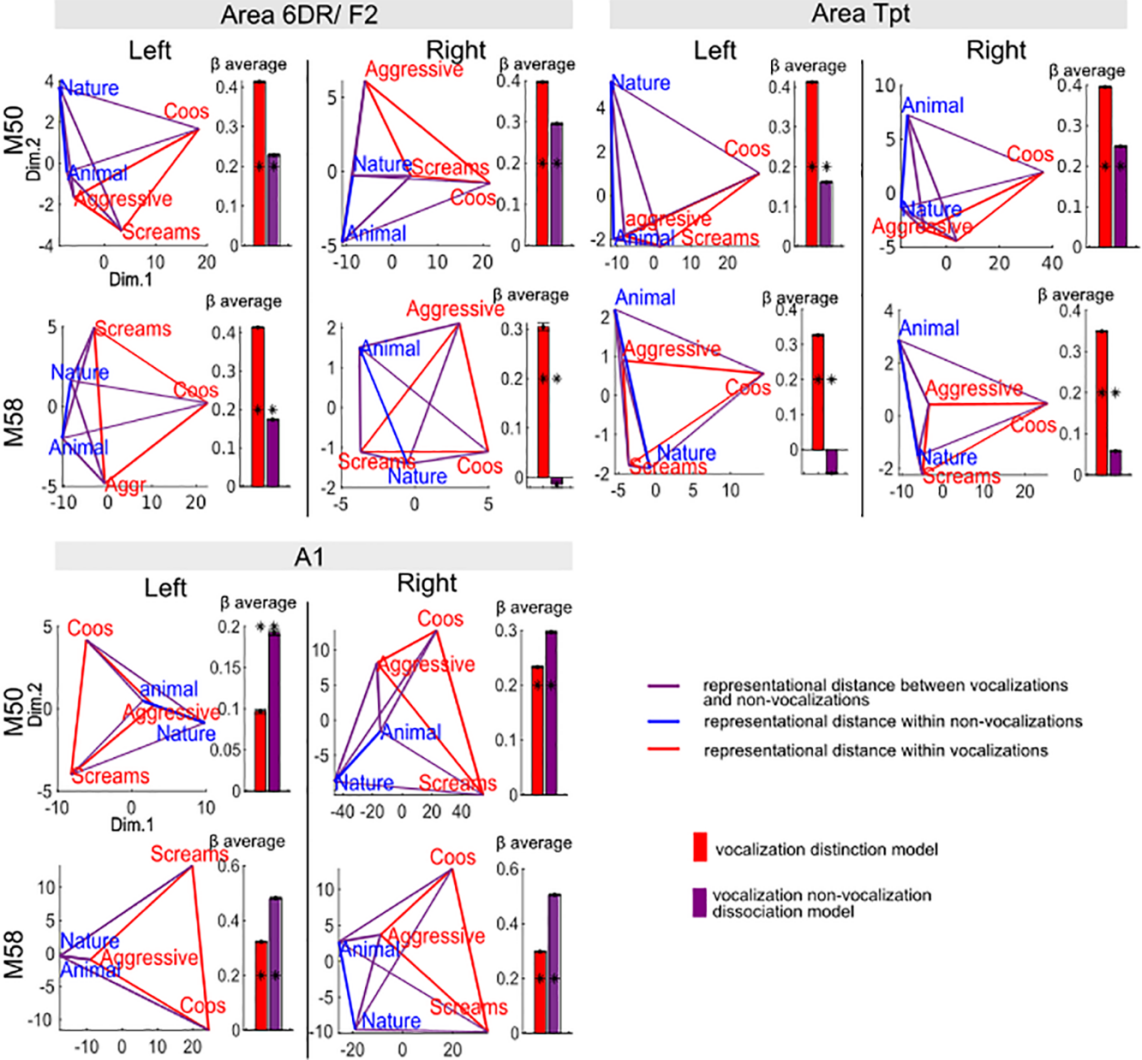
Representational geometry of different sound categories within each region of interest for both monkeys. Left side of each panel: 2D visualization of the representational geometry in each region. An average DSM was estimated using a repeated hold-out method and embedded in 2D space through non-classical DSM. The Red/Blue lines indicate distances within the vocalization and non-vocalization categories. The purple lines represent the distance between vocalizations and non-vocalizations. The thickness of the lines corresponds to the normalized similarity in its original space. Right side of each panel: the model fits DSM by a multiple regression RSA, representing the strength of certain coding strategies. The bars show the β average obtained from the repeated hold-out method, with error bar representing confidence intervals. Red bars correspond to the vocalization distinction model where a positive value indicates higher dissimilarity between vocalizations compared to non-vocalizations, and a negative value indicates the opposite. Purple bars are for the vocalization/non-vocalization dissociation model, in which a positive value means a higher dissimilarity between vocalization and non-vocalization, than within each group. The stars represent βs significantly different from zero (*p < 0.05). Results are shown for both M50 and M58.

To examine the representational geometry of auditory coding, we conducted a multiple regression RSA (Fig. 9, and Supplementary Fig. S6E for area R). Both area 6DR and Tpt exhibited greater dissimilarity between the three vocalizations compared to the non-vocalization types, indicating a stronger capacity to discriminate information between different vocalization types than between different non-vocalization types. In most instances, these regions, along with A1, showed a clear distinction between vocalizations and non-vocalizations (positive purple bars), except for the right hemisphere in M58. The results for area R were more variable across monkeys, reflecting the findings from the percent signal change analyses. One monkey (M50) exhibited greater dissimilarity among non-vocalizations than vocalizations (M50), while M58 showed less dissimilarity within and between vocalizations and non-vocalizations.

Combining the results of the RSA with the percent signal change analysis, A1 appears to be responsive to all the sounds and it can discriminate between them. In contrast, areas 6DR and Tpt respond specifically to vocalizations and encode different types of vocalizations.

Although vocalizations did not evoke higher activity than scrambled vocalizations in Tpt and 6DR (except for M50; Fig. 6), 3-class MVPA for both ROIs indicates that these areas can discriminate these stimuli based on the pattern of activity of their voxels (Fig. 7).

Contrasting the vocalizations with their scrambled versions did not reveal major differences in brain activation. We do not show the empty maps obtained from this contrast but Supplementary Figure S3A shows the activation map of scrambled vocalizations versus silence which is almost identical to the vocalizations versus silence map (Fig. 5A). In addition to showing the contrasts between vocalizations and non-vocalizations for both monkeys combined, we provide activation maps of each condition versus silence in Supplementary Figures S4 and S5. Specifically, these maps display the activations for each vocalization and scrambled vocalization versus silence, as well as each non-vocalization and scrambled non-vocalization versus silence.

The 3-class MVPA analysis, however, revealed that the ROIs within areas 6DR and Tpt can discriminate the different types of vocalizations but not the scrambled vocalizations. Thus, only the pattern of activity and not the absolute activity levels in these areas can distinguish between vocalizations and scrambled vocalizations.

In Supplementary Figure S3B, we displayed the activation for non-vocalizations versus silence (see also Supplementary Fig. S5 for animal and nature versus silence separately). This map shows activations extending to the most anterior part of the temporal lobe, including the rSTG where the anterior temporal vocalization area (TVa) is located according to previous studies (Bodin et al., 2021; Petkov et al., 2008). This result indicates that this anterior portion of the lateral sulcus can be activated by auditory stimuli. Surprisingly, however, we did not observe a difference between vocalizations and non-vocalizations.

To investigate a little further the anterior part of the lateral sulcus, we selected the most rostral ROI in this sulcus—that is, within area R—which was the closest to reach our criteria to be vocalization selective. We performed the same ROI analysis as for the three precedent ROIs (Supplementary Fig. S6). This ROI was activated by all three sound categories in M58 (p < 0.001). In M50, activation was observed only for vocalizations and scrambled vocalizations (p < 0.001). Vocalizations elicited higher activation than non-vocalizations in both hemispheres of both monkeys (p < 0.001; Supplementary Fig. S6B). Notably, only the pattern of activity of the voxels in right area R of M58 significantly discriminated between the three types of vocalizations. Moreover, none of the four R ROIs were able to differentiate between the vocalizations and scrambled vocalizations (Supplementary Fig. S6C). Investigating the percent signal change for each condition, the results were variable between the two monkeys (see Supplementary Fig. S6D and Supplementary Table 7 for the p values). In M58’s R area, screams elicited higher activation than the other sounds, while nature sounds and their scrambled versions produced generally lower fMRI responses. In contrast, M50 was not activated by animal calls in both hemispheres, with stronger activation for coos than non-vocalizations in the right hemisphere. The ability to classify stimuli based on the pattern of activity in area R was unclear (Supplementary Fig. S6E). However, this region seems to differentiate screams more distinctly, while the other ROIs identify the coos as more dissimilar than the other sounds.

In Figure 5B and Table 2, we can observe that other regions than Tpt and 6DR reach the first criteria of ROI selection. This is the case in areas such as SII, Ig, and PEa. Area 8B also shows significant group-level activations at 0.05 FWE correction but not with a cluster correction of 10 voxels. These areas, however, did not meet our stringent individual-level criterion (p < 0.001 uncorrected in each monkey and hemisphere separately) based on half of the data which were used to create the respective ROIs, and therefore they were not selected as primary ROIs. However, to be as comprehensive as possible and because the involvement of these regions would make sense in a network involved in processing complex vocalizations and linking auditory input to motor system, we also provide a supplementary figure showing the percent signal changes for each condition in these areas. ROIs were created with the combined data of both monkeys in MEBRAINS template space (Supplementary Fig. S7; half of the combined data being used for selecting the ROIs and the other half for the ROI analysis). PEa’s ROI shows significant activation for aggressive calls, coos, scrambled coos, and screams in each hemisphere in the first and the second half of the data (Wilcoxon nonparametric test, p < 0.001 and p < 0.01, respectively). All vocalizations and scrambled vocalizations elicit a significant response in the granular insula and SII (Wilcoxon nonparametric test p < 0.001; both datasets and hemispheres), except for the scrambled screams which evoked no activations in SII of the right hemisphere in neither halve of the dataset. Animal calls and nature sounds, as well as their scrambled versions, produce no significant activations, or even deactivate PEa, and Ig. As for all the data, scrambled animal calls provoke a response in SII in 3 hemispheres out of 4. Area 8B was more variable, as it shows activations for aggressive calls and coos in 3 out of 4 hemispheres; scrambled coos and screams in 4 /4 hemispheres; animal calls in 1/4; scrambled animal calls in 2/4; and nature sounds and scrambled nature sounds in 3/4 hemispheres, with no significant difference between these conditions.

## 4 Discussion

### 4.1 Monkey ventral and dorsal vocalization networks

In this paper, we show a large network responsive to vocalizations in two monkeys, which may be subdivided into a ventral and a dorsal stream as suggested earlier (Kusmierek & Rauschecker, 2014; Rauschecker & Scott, 2009; Rauschecker & Tian, 2000; Romanski & Averbeck, 2009). Our results indicate that the putative ventral stream includes vocalization-responsive activations in the rostral part of the STG, the insula, area 6 V (F5), and areas 45A within ventrolateral prefrontal cortex. In contrast, regions potentially belonging to the dorsal stream encompass sectors within the caudal STG, the dorsal premotor cortex, area 8a, and dorsolateral prefrontal area 46D. Subcortically, we identified the inferior colliculus and the medial pulvinar, both known components of the auditory system that respond to vocalizations (Baumann et al., 2011; Froesel et al., 2021, 2024; Kaas & Hackett, 1998). Additionally, our results showed vocalization responses in primary visual cortex (V1) and higher-order visual areas, including V4 and TE. This supports previous findings from PET (Gil-da-Costa et al., 2004; Poremba et al., 2003) and fMRI studies (Froesel et al., 2022). Furthermore, we report activations in somatosensory area SII, consistent with earlier studies (Bodin et al., 2021), as well as in area 24c’ of the cingulate cortex, which has not been previously documented. Certain regions within this network exhibit selective activations for vocalizations compared to non-vocalizations. This was the case in areas Tpt, 6DR/F2 as well as SII, granular insula, which are involved in somatosensory processing (Evrard, 2019), and area PEa and 8B (when the data of both monkeys were combined, see Figure 5 and Supplementary Fig. S4). This network, encompassing auditory-associated, premotor, and somatosensory-related areas, is consistent with the concept of a dorsal pathway potentially involved in linking auditory input to motor systems or action representations in macaques (Rauschecker, 2018). However, to gain a full understanding of this sound-to-action mapping in macaques for simple vocalizations, particularly in the context of a passive listening task and with concatenated stimuli, further studies may be required.

### 4.2 Temporal voice area and putative macaque homologs of human TVa voice patches

By comparing vocalizations with non-vocalizations, we identified a region in the rostral division of the auditory cortex. At first sight, it may seem appropriate to associate this activation with the monkey counterpart of the human temporal vocal area (TVa), as previously reported (Bodin et al., 2021; Petkov et al., 2008). However, the region we found is located more posteriorly and caudally than the proposed monkey homolog of TVa. Specifically, it falls within the rostral core region of the auditory cortex, when mapped on the D99 and CHARM atlases, while Bodin and colleagues’ voice-selective TVa was situated in areas rSTG and the rostral parabelt area (RPB). Several factors could explain these apparent differences. First, similar to face patches, the precise location of category-selective regions may vary depending on the subject and can differ between hemispheres (Janssens et al., 2014; Vanduffel et al., 2014). Second, the activation we observed might represent a voice-sensitive region in the macaque auditory cortex that is distinct from the anterior TVa homologue proposed by Bodin et al. (2021) and Petkov et al. (2008). Its location could potentially make it analogous to the human mid-TVa (mTVa), assuming a similar multi-patch organization exists in macaques (Pernet et al., 2015). Notably, also other macaque studies did not identify an anterior region, although they did show preferential activations in a more caudal part of the lateral sulcus, closer to our vocalization-specific activation (Joly, Ramus, et al., 2012; Ortiz-Rios et al., 2015). One might also argue that lower signal-to-noise ratios may hinder our ability to identify the homolog of the aTVa. However, the signal-to-noise ratios were not substantially different—or lower—between RPB (M50: left: 11.6, right: 28; M58: left:28.1, right:32.1) and rSTG (M50: left:13.6, right:21.76; M58: left:20.9, right:32.7) compared to the other regions such as the rostral area R of the core region of auditory cortex (M50: left: 14, right: 21; M58: left:18.3, right:18.6) (see Supplementary Fig. S8). Moreover, we observed several reproducible bilateral vocalization-specific activations in both monkeys, including regions previously identified as preferring conspecific vocalizations (Petkov et al., 2008) reinforcing the notion that signal-to-noise ratio does not explain the discrepancies between our findings and previous studies. A more parsimonious explanation could be that differences in task design, acquisition parameters, or specific stimuli used across studies account for the variations in descriptions of some voice patches.

Overall, if we assume that the conspecific vocalization-preferring clusters we identified represent the macaque homolog of mid-TVa, then the macaque voice patch network might consist of three distinct clusters: a posterior patch near A1, and a middle and anterior patch. This aligns with the suggestion that there could be multiple interconnected voice patches in the macaque brain, similar to those reported in humans (Belin et al., 2018). Electrophysiological studies will be essential to confirm this hypothesis by identifying voice selective neurons within these proposed voice patches, as previously demonstrated in the anterior macaque patch by Perrodin et al. (2011) and more recently by Giamundo et al. (2024).

### 4.3 Dorsal premotor area and temporal area Tpt: nodes of a dorsal auditory-motor network?

The contrast between vocalizations and non-vocalizations yielded robust activations in regions that were either weakly activated or rarely reported in previous fMRI or PET auditory studies (Gil-da-Costa et al., 2006), notably within the caudal temporal lobe (area Tpt) and dorsal premotor cortex (area 6DR/F2).

The first notable activation is located in the caudal part of the temporal lobe within the temporo-parietal region, which corresponds to cytoarchitectonic area Tpt, as identified in the D99, CHARM and CIVM atlases. Even in their PET study, Gil-da-Costa et al. (2006) noted that the caudal part of the STG, also referred to as Tpt, exhibits stronger responses to vocalizations compared to other sounds. Yet, this region is rarely described as vocalization responsive or selective. Our percent signal change and MVPA analyses revealed a strong sensitivity to vocalizations in Tpt, and a notable ability to distinguish between different types of vocalizations versus their scrambled versions. Based on its vocalization-selective responses, Tpt has been proposed as a potential macaque analogue to Wernicke’s area in humans (Gil-da-Costa et al., 2006), a region well-known for its crucial role in language processing. Our findings of strong vocalization sensitivity and discriminability in macaque Tpt further support the notion that this area is a key node in the primate vocal processing network, potentially analogous to temporal regions in humans involved in processing complex auditory signals, including vocal communication. However, it is important to note that while Wernicke’s area in humans is critical for language comprehension and production (Damasio & Damasio, 1980), our data solely demonstrate Tpt’s involvement in vocalization perception and do not provide evidence for a putative role in vocalization production.

This brings us to another area that showed higher responses to vocalizations than to non-vocalizations: the dorsal part of premotor area 6 (6DR). Also known as area F2, this region demonstrates strong sensitivity to vocalizations and a greater capacity to discriminate among the three vocalizations compared to their scrambled counterparts. Area F2 is a part of the dorso-medial stream, which is involved in reaching and is associated with visual areas and trunk/hindlimb motor areas (Greulich et al., 2020). Functional connectivity analyses demonstrated that F2 activity correlates not only with that in V6A and MIP but also with that in area Tpt, the insula, SII, the rostral part of the auditory cortex, and areas 44, 45, and 46, as well as the medial pulvinar and the claustrum (Greulich et al., 2020). This extensive functionally connected network suggests that, beyond reaching, this area may also play a role in higher-level auditory-related functions. This network presents high similarities with the putative dorsal auditory network described in the first paragraph which we observed when combining the data of the two monkeys (Fig. 5B).

During our experiment, monkeys were required to keep their hands on a bar and fixate on the screen. Since we only included runs where the monkey reached > 90% fixation accuracy without hand movements, it is unlikely that the observed activation in 6DR/F2 is caused by these behaviors. Furthermore, the number of rewards, and consequently the licking or sucking movements to obtain juice did not differ across conditions (Nonparametric Friedman test: M58: X^2^: 20.36, p = 0.4; M50: X^2^: 7.41, p = 0.99). Hence, it is improbable that the vocalization-specific activations in 6DR/F2 were driven by non-auditory factors, strongly supporting its role in the vocalization perception network.

A study in which macaques learned an auditory–motor task—producing sounds by pressing levers—demonstrated that auditory melodies, once associated with movement, activate not only the auditory cortex but also motor and premotor areas. This was particularly the case for F2, a region associated with arm and reaching-related activity (Archakov et al., 2020). This activation was expected, as the monkeys had learned to associate melodies with arm movements. Based on this finding, one might hypothesize that vocalization-related activity in the premotor cortex would be confined to ventral regions such as 6 V, which is linked to orofacial movements including vocalization production (Coudé et al., 2011; Ferrari et al., 2003). However, while vocalization-related activity was observed when contrasting vocalizations with silence (Fig. 5A), no difference in 6 V activity was found when contrasting vocalizations versus non-vocalizations (Fig. 5B). This contrasts to the results observed in dorsal premotor cortex. While this dorsal premotor activation does not fit with the original hypothesis, it is consistent with recent comparative evidence. Specifically, fMRI studies in marmosets, which have a more complex vocal repertoire, also report increased activation in the homologue of macaque area 6DR during vocalization processing, even when the animals were not producing calls (Dureux et al., 2024; Jafari et al., 2023). Electrophysiological recordings in marmoset 6DR have further documented neurons responsive to both vocal signals and vocal motor production (Miller et al., 2015). These findings in marmosets, together with our macaque data during passive listening, suggest that 6DR/F2 is involved in the perception of vocalizations in primates, potentially as part of an auditory-motor integration system that is not exclusively tied to overt production or specific effectors. In humans, area 6 is also activated when subjects listened to speech (Wilson et al., 2004). A recent electrophysiological study reported single-neuron and population-level semantic encoding during language comprehension in the human dorsal prefrontal cortex overlapping with area 6 (Tang et al., 2023). Our multivoxel pattern analysis indicates 6DR/F2 and Tpt are specifically responsive to vocalizations, which they can discriminate from non-vocalizations. Moreover, these areas encode the different types of vocalizations.

## 5 Discussion on Acoustic Content and Regional Specificity

The differential responses observed across auditory regions likely reflect varying sensitivities to specific acoustic features of the sound categories (Table 1, Fig. 2). Our results indicate that, while primary auditory cortex (A1) responded to vocalizations, regions such as Tpt and 6DR exhibited higher selectivity and discriminability for vocalizations (Figs. 6 and 7).

Specifically, screams elicited strong responses in A1, consistent with their broadband, high-frequency profile, and features typically processed by early auditory areas. Screams are characterized by the highest spectral centroid, a high average F0, and elevated pitch variability (F0 SD). However, their HNR was intermediate, lower than coos but higher than aggressive and nature sounds. In contrast, coos, with their more tonal and structured acoustic pattern (reflected by higher HNR and lower F0 variability), evoked robust and discriminative responses in Tpt and 6DR. This suggests that these higher-level regions may be particularly sensitive to the stable spectral patterns and social vocal cues present in coos.

Additionally, our stimulus design, which involved concatenating short vocal elements within each block, introduced artificial temporal regularities that do not reflect the natural temporal structure of macaque vocal exchanges. This compromise was necessary for the blocked fMRI design, which requires sustained input to generate robust BOLD/MION signals. However, while macaques do not produce long, continuous sequences, they engage in rapid, overlapping vocal exchanges during social interactions, which our multi-exemplar blocks partially capture.

Crucially, individual vocalizations were presented in their entirety and unaltered, preserving their natural spectro-temporal features. Since non-vocal sounds were presented with the same block structure, the observed selectivity in area Tpt and 6DR likely reflects sensitivity to the acoustic and social characteristics of vocalizations, rather than their temporal structure. This supports the idea that these regions are involved in processing complex or socially relevant auditory signals.

As highlighted previously, contrasting the vocalizations with their scrambled versions did not reveal major differences in brain activation. To further examine the nature of the observed vocalization selectivity, we additionally generated the contrast between scrambled vocalizations and non-vocalizations (Supplementary Fig. S9). This contrast reveals that most regions showing stronger activation for vocalizations compared to non-vocalizations also respond more strongly to scrambled vocalizations than to non-vocalizations. Interestingly, however, this is not the case for area 6DR. Since scrambled vocalizations preserve many low-level acoustic features, they may still be perceived by the monkeys as biologically relevant or vocal-like sounds, somewhat limiting their effectiveness as control stimuli. The lack of differential activation between natural and scrambled vocalizations likely reflects this perceptual similarity rather than an absence of cortical specificity for vocalizations. Supporting this interpretation, our 3-class MVPA analyses revealed that both 6DR and Tpt exhibited above-chance classification accuracy for distinguishing between different types of natural vocalizations, with accuracy notably higher than for classifying scrambled vocalizations. Together, these findings suggest that regions such as 6DR and Tpt are selectively tuned to features characteristic of natural vocal output, including coherent articulatory structure and socially meaningful acoustic patterns. This supports the interpretation that these areas contribute to processing complex, species-specific vocalizations, rather than responding indiscriminately to all acoustically rich stimuli.

Our study includes a male and female. While our study did not specifically explore the effects of sex on vocalization perception, due to our small sample size, these factors warrant consideration as they can potentially influence behavioral and neural responses. For instance, at the behavioral level, female monkeys have been shown to produce more food-related calls and exhibit greater responsiveness to copulation calls than males (Hauser, 2007; Hauser & Marler, 1993). Conversely, another study suggests higher male performance in auditory memory tasks involving animals, humans, and monkeys, as well as natural sounds (Ng et al., 2009).

In humans, language processing is thought to be organized into dorsal and ventral pathways (Hickok & Poeppel, 2004, 2007), with the dorsal stream playing a crucial role in sensorimotor integration and sound-to-action mapping, essential for speech processing (Rauschecker & Scott, 2009; Saur et al., 2008). Anatomical studies in primates have identified major white matter tracts like the arcuate fasciculus (AF) and superior longitudinal fasciculus (SLF), which connect posterior temporal regions, such as area Tpt, with frontal areas, including dorsal premotor cortex (6DR/F2) and prefrontal cortex (Petrides & Pandya, 1988; Schmahmann & Pandya, 2006). While some frameworks, based on anatomical tracing, have proposed specific dorsal circuits linking inferior parietal and premotor areas (including those controlling orofacial musculature) to area 44 as a potential precursor circuit in macaques (Petrides & Pandya, 2009), the functional roles of these pathways in macaque vocal communication remain an active area of research.

Based on the results showing activation within Tpt and 6DR, along with other areas involved in voice processing, such as the intraparietal area PEa, the granular insula, SII and 8B (Supplementary Fig. S4), we hypothesize that these regions form a dorsal voice-related network. This dorsal pathway for vocalization processing, anatomically present in primates, may be inherited from a common ancestor. It could have given rise to the specific circuitry supporting vocalization processing in monkeys and the more elaborate pathways involved in human speech perception and production.

## 6 Conclusion

Using submillimeter fMRI, we investigated the network associated with conspecific vocalizations in macaques. Our findings reveal a dorsal network involving Tpt and a portion of 6DR, which show a preference for vocalizations over other sound categories and can effectively discriminate between different types of vocalizations, such as coos, screams, and aggressive calls. These results highlight the involvement of a dorsal pathway in macaque vocalization processing and contribute to our understanding of the neural basis of vocal communication in primates, potentially offering insights into the evolutionary precursors of auditory-motor pathways supporting complex auditory behaviors, including human speech.

## Supplementary Material

Supplementary Material

## Data Availability

The data and code are available via KU Leuven RDR dataset named High-resolution fMRI that reveals a dorsal brain pathway selective for conspecific vocalizations in macaques (https://doi.org/10.48804/P841JQ). Any additional information required to reanalyze the data reported in this paper is available from the lead contacts upon request.
