# Supplementary material for: High-resolution fMRI reveals a dorsal brain pathway selective for conspecific vocalizations in macaques
Source: Imaging Neurosci (Camb). 2025 Aug 13;3:IMAG.a.108. doi: 10.1162/IMAG.a.108 (PMC12351310; doi:10.1162/IMAG.a.108)
Supplement: Supplementary Material [file IMAG.a.108_supp.pdf]

Supplementary data

Table S1. vocalizations > silence and vocalizations > non-vocalizations maxima of M50. Related to Figure 2 and 3. **bold**: $P < 0.05$ FWE corrected, t-score> 5.1. $P < 0.05$ FDR corrected, t-score> 4.43; P<0.001 uncorrected, t-scores> 3.09; p<0.005 uncorrected, T>2.58. In **bold** the label of the region selected for the ROI analysis. SII: secondary somatosensory cortex; Ig: granular insula; Id: dysgranular insula; Ri (retroinsula); CM: caudomedial belt region; A1: primary auditory cortex; Tpt: temporoparietal area; CL: caudolateral belt region; AL: anterior lateral belt; R: rostral core region; RM: rostro medial region; RTM: rostrotemporal belt; rSTG: rostral superior temporal sulcus; MST: medial superior temporal area; V4t: dorsal V4); VIP: ventral intraparietal area; MGN: medial geniculate nucleus ; IC: Inferior colliculus; SC Superior colliculus; pulvinar: Pul; Oct: olivo cerebellar tract.

**M50 (monkey space)**					
Label D99 (CHARM 6)	Peak coordinates (mm) (origin: anterior commissure)			T scores vocalizations vs silence	T scores vocalizations vs non-vocalizations
L 46D	-25	-45	28	**9.4**	-1
R 46D	27	-44	30	**6.5**	-0.2
R 9m	3	-49	38	**8.35**	0.2
**L 6DR**	-19	-11	54	**10.61**	**7.62**
**R 6DR**	14	-8	-43	**8.39**	**5.13**
L F5/6V	-39	-8	32	**5.39**	3.79
R F5/6V	34	-5	30	**5.76**	4.73
L 45a	-46	-38	20	**13.64**	-1.1
R 45a	51	-31	21	**10.44**	3.4
L 45b	-50	-24	23	3.64	1.6
R 45b	45	-18	22	4.2	1.55
L SII	-52	-4	15	**12.63**	**7.07**
R SII	52	-1	12	**12.02**	**7.98**
L Ig	-45	7	16	**9.8**	**5.7**
R Ig	42	9	18	**7.4**	**5.5**
L Id	-51	-4	-4	2.22	3.69
R Id	48	-3	-1	4.23	2.9
L Ri	-37	30	22	**6.9**	3.7
R Ri	38	26	14	**8.9**	**6.3**

L CM	-38	35	18	**10.33**	**5.23**
R CM	35	35	15	**12.09**	4.9
**L A1**	-51	35	22	**13.71**	**5.88**
**R A1**	46	40	35	**18.52**	**6.69**
**L Tpt**	-52	49	36	**10**	**7.4**
**R Tpt**	39	53	32	**9.9**	**6.7**
L CL	-59	34	25	**13.21**	**5.29**
R CL	58	46	30	**9.7**	4.3
L AL	-61	27	18	**8.2**	3.4
R AL	61	131	19	**11.36**	4.57
**L R**	-56	19	25	**11.30**	3.09
**R R**	52	20	27	**9.4**	3.3
L RM	-57	11	-10	**9.34**	1.44
R RM	52	5	-14	**7.2**	1.58
L RTM	-54	-5	-17	**7.07**	-0.2
R RTM	54	0	-314	**5.6**	0.8
L rSTG	-65	6	-41	**7.58**	-0.88
R rSTG	62	6	-3	**11.15**	0.16
L TAa	-64	10	-21	1.96	3.09
R TAa	67	11	-18	1.51	2.79
L TPO	-61	32	4	3.12	1.6
R TPO	56	23	-8	**5.4**	4.3
L IPa	-47	30	-8	1.02	2.8
R IPa	48	36	-9	4.6	3.1
L MST	-41	51	31	4.2	3.6
R MST	42	58	30	5.01	3.55
L area 3	-39	34	56	**5.69**	2.6
R area 3	22	34	57	4.6	2.6
L area 1-2	-39	35	56	3.7	2.8
R area 1-2	32	39	57	4.1	2.7
L 24c	-4	-3	39	4.42	3.88

R 24c	9	15	37	4.5	3.6
L F1	-7	18	45	4.3	2.6
R F1	7	21	44	**5.9**	4.03
L V4t	-51	68	33	3.25	3.6
R V4t	52	64	30	4.46	3.6
L V1	-55	89	13	3.7	1.17
R V1	50	90	6	4.7	1.32
L PEa (5)	-36	35	43	3.023	3.6
R PEa (5)	26	52	50	3.11	2.05
L VIP	-24	43	34	2.57	2.59
R VIP	27	42	39	2.54	2.46
Subcortical (SARM)					
L IC	-10	45	-12	3.09	1.9
R IC	7	43	-12	3.34	1.2
L SC					
R SC	8	43	-2	2.63	1.71
L MGN	-22	30	-11	2.78	1.9
R MGN	22	32	-7	2.78	0.2
L medial Pul					
R medial Pul	14	37	10	3.2	1.9
L lateral Pul					
R lateral Pul					
L Oct	-16	67	-20	2.1	3.9
R Oct	15	61	-24	2.3	0.43
L claustrum	-47	7	-10	4.27	2.8
R claustrum	42	9	-13	3.7	2.2

Table S2. vocalizations > silence, and vocalizations > non-vocalizations maxima of M58. Related to Figure 2 and 3. **bold**: *P* < 0.05 FWE corrected, t-score> 5.1. *P* < 0.05 FDR corrected, t-score> 4.43; P<0.001 uncorrected, t-scores> 3.09; p<0.005 uncorrected, t-score >2.58. In **bold** the label of region selected for the ROI analysis. SII: secondary somatosensory cortex; Ig: granular insula; Id: dysgranular insula; Ri (retroinsula); CM: caudomedial belt region; A1: primary auditory cortex; Tpt: temporoparietal area; CL: caudolateral belt region; AL: anterior lateral belt; R: rostral core region; RM: rostro medial region; RTM: rostrotemporal belt; rSTG: rostral superior temporal sulcus; MST: medial superior temporal area; V4t: dorsal V4); VIP: ventral intraparietal area; MGN: medial geniculate nucleus ; IC: Inferior colliculus; SC Superior colliculus; pulvinar: Pul; Oct: olivo cerebellar tract.

**M58 (monkey space)**					
Label D99 (CHARM 6)	Peak coordinates (mm) (Origin: anterior commissure)			T scores vocalizations vs silence	T scores vocalizations vs non-vocalizations
L 46D	-18	-54	27	**8.25**	2.5
R 46D	26	-36	31	3.7	2.7
L 8B	-19	-30	48	**6.4**	**5.11**
R 8B	18	-28	50	**13.78**	**5.39**
**L 6DR**	-14	-13	52	**6.9**	3.8
**R 6DR**	11	-17	63	4.08	3.32
L F5/6V	-36	-5	25	4.59	2.58
R F5/6V	36	-3	31	3.2	2.6
L 45a	-54	-33	13	**8.5**	0.9
R 45a	53	-29	17	**6.1**	3.04
L 45b	-46	-24	12	4.9	3.1
R 45b	39	-14	20	3.3	2
L 12m	-39	-42	15	4.8	2.7
R 12m	36	-37	19	**6.4**	3.2
L 13l	-39	-34	13	**7**	0.006
R 13l	37	-34	13	**8.5**	0.5
L SII	-57	23	9	**10.3**	2.7
R SII	60	24	12	**6.7**	3.06
L Ig	-42	11	14	**8.4**	3.5
R Ig	42	17	16	**7.6**	4.2
L Id	-51	11	-2	**6.2**	2.2
R Id	53	11	1	5.03	3.5

L Ri	-36	38	17	**7.1**	2.5
R Ri	35	36	18	**10**	4.7
L CM	-35	39	14	**10.7**	**5.25**
R CM	36	39	15	**17**	**6.6**
**L A1**	-44	42	14	**24.9**	**6.3**
**R A1**	51	38	15	**15.5**	**6.5**
**L Tpt**	-54	52	24	**10.21**	4.78
**R Tpt**	42	52	27	**12**	**6.74**
L CL	-58	50	20	**18.9**	**7.3**
R CL	55	47	24	**11.8**	4.5
L AL	-62	14	-2	**18.7**	3.4
R AL	60	16	-2	**17**	**5.4**
L R	-59	16	-4	**10**	3.08
R R	57	14	-4	**19.9**	**5.7**
L RM	-53	13	-10	3.4	1.46
R RM	52	1	-13	1.3	1.2
L RTM	-63	-2	-5	**23.5**	0.16
R RTM	60	-2	-7	**11.5**	3.1
L rSTG	-65	2	-18	**5.8**	0.5
R rSTG	70	4	-15	3.39	1.05
L TAa	-65	7	-17	4.1	1.8
R TAa	67	7	-15	3.46	0.3
L TPO	-61	34	0	**5.7**	2
R TPO	56	31	-6	**9.9**	2.38
L IPa	-53	34	-16	3.7	1.4
R IPa	56	30	-20	3.6	0.5
L MST	-33	59	19	1.8	1.2
R MST	41	59	23	3.6	2.2
L area 3	-40	26	33	3.08	0.88
R area 3	35	22	32	4.6	2.5
L area 1-2	-51	25	39	3.1	1.07

R area 1-2	52	14	35	**6.9**	3.2
L 24c	-13	21	38	4.4	2.08
R 24c	10	15	33	4.7	2.1
L F1	-15	21	39	**6.2**	3.7
R F1	11	24	38	**5.2**	3.36
L V4	-49	77	16	3.5	1.24
R V4	48	73	21	3.3	2
L V1	-19	98	-2	3.5	-0.1
R V1	31	108	7	3.4	1.6
L PEa (5)	-33	49	44	4.18	1.2
R PEa (5)	34	41	40	**5.6**	2.7
L VIP	-34	36	32	3.9	1.4
R VIP	28	39	32	3.5	1.1
Subcortical (SARM)					
L IC	-6	48	-13	**5.4**	2.7
R IC	10	47	-15	3.2	2.57
L SC	-1	50	-4	2.8	2.1
R SC	3	50	-5	3.53	2.24
L MGN	-18	35	-5	2.64	1.4
R MGN	23	35	-10	2.7	1.36
L medial Pul					
R medial Pul	13	33	21	3.2	2.7
L lateral Pul	-29	38	4	2.8	2.4
R lateral Pul	24	38	7	2.02	3.7
L Oct	-10	61	-24	2.6	0.35
R Oct	14	60	-28	2.8	1.4
L claustrum	-33	-3	-18	3.5	1.8
R claustrum	34	-6	-22	2.6	0.8

Table S3. vocalizations > silence, and vocalizations > non-vocalizations maxima of Combined monkeys (MEBRAINS space). Related to Figure 2 and 3. **bold**: *P* < 0.05 FWE corrected, t-score> 5.1. *P* < 0.05 FDR corrected, t-score> 4.43; P<0.001 uncorrected, t-scores> 3.09; p<0.005 uncorrected, t-score >2.58. In **bold** the label of region selected for the ROI analysis. SII: secondary somatosensory cortex; Ig: granular insula; Id: dysgranular insula; Ri: retroinsula; CM: caudomedial belt region; A1: primary auditory cortex; Tpt: temporoparietal area; CL: caudolateral belt region; AL: anterior lateral belt; R: rostral core region; RM: rostro medial region; RTM: rostrotemporal belt; rSTG: rostral superior temporal sulcus; MST: medial superior temporal area; V4t: dorsal V4); VIP: ventral intraparietal area; MGN: medial geniculate nucleus ; IC: Inferior colliculus; SC Superior colliculus; pulvinar: Pul; Oct: olivo cerebellar tract.

**Combined monkeys (MEBRAINS space)**					
Label D99 (CHARM 6)	Peak coordinates (mm)			T scores vocalizations vs silence	T scores vocalizations vs non-vocalizations (voxel counts (kE))
L 46D	-16	44	43	**6.9**	-0.07
R 46D	26	32	44	7	0.5
L 8B	-19	19	61	**8.6**	4.7 (4vox)
R 8B	18	17	64	**15**	3.96 (22 vox)
**L 6DR**	-19	-4	65	**9.3**	**7.7 (24 vox)**
**R 6DR**	20	-3.6	63	**9.1**	**6.1 (16 vox)**
L F5/6V	-36	-5	43	**9.7**	2.58
R F5/6V	41	-4	45	5.6	2.1
L 45a	-52	22	29	**11.2**	2.06
R 45a	54	22	30	**17.1**	3.8 (62 vox)
L 45b	-46	13	37	3.3	0.9
R 45b	44	11	38	**9.9**	2.06
L 12m	-37	22	31	5.9	1
R 12m	36	22	30	**9.2**	3.05
L 12l	-44	26	34	**13.2**	1.54
R 12l	56	19	29	**8.6**	2.4
R 13l	31	16	27	3.2	1.14
L SII	-50	-10	28	**9.5**	**6.08 (34 vox)**
R SII	53	-8	28	**10.65**	**7.9 (50 vox)**

L Ig	-41	-36	26	**11.02**	**6.8 (20 vox)**
R Ig	40	-38	27	**16.18**	**8.3 (23 vox)**
L Id	-49	-23	14	**5.2**	1.8
R Id	48	-29	20	**7.8**	2.05
L Ri	-45	-42	36	**7.9**	4.08 **(6 vox)**
R Ri	35	-44	34	**7.7**	2.4
L CM	-44	-48	30	**27.8**	**8.7 (24 vox)**
R CM	37	-47	32	**6.04**	**5.3 (41 vox)**
**L A1**	-47	-48	34	**54**	**8.6 (97 vox)**
**R A1**	47	-50	32	**47.7**	**10.7 (139 vox)**
**L Tpt**	-47	-65	47	**10.2**	**6.65 (48 vox)**
**R Tpt**	41	-61	45	**7.8**	**5.3 (44 vox)**
L CL	-56	-50	32	**26.6**	**6.83 (11 vox)**
R CL	59	-49	33	**23.7**	4.9 **(18 vox)**
L AL	-70	-23	22	**47.4**	4.9 (20 vox)
R AL	63	-29	22	**57.2**	**7.2 (10 vox)**
L R	-59	-26	15	**16.8**	3.8 (25 vox)
R R	58	-27	18	**14.8**	4.6 (10vox)
L RM	-55	-23	5	10	1.26
R RM	57	-17	6.9	10.29	1.08
L RTM	-56	-15	2.9	4.5	1.9
R RTM	53	-12	2.8	4.6	0.4
L RTL	-5.9	-5	7.9	**7.6**	-2.21
R RTL	59	-4.1	7.9	**10.88**	-1.37
L rSTG	-60	-6.1	10	**7.4**	2.1
R rSTG	59	-4.7	6.8	**12.9**	-1.9
L TAa	-55	-34	6.9	3.3	0.1
R TAa	59	-32	6.9	**6.1**	3.3
L TPO	-56	-31	3.9	3.52	0.33
R TPO	57	-31	6.9	**5.5**	3.2
L MST	-40	-68	42	3.2	4.3

R MST	42	-71	42	5.3	1.8
L TE	-67	-36	7.4	6.7	2
R TE	70	-35	7.4	6.7	0.2
L area 1-2	-28	-48	71	8.2	2.7
R area 1-2	33	-48	67	**9.4**	0.3
L 24c’	-4	-9.6	51	6.9	4.3 (28 vox)
R 24c’	6	-15	50	4	2.5
L V4	-55	-79	40	2.1	0.4
R V4	49	-79	48	3.8	1.24
L V1	-28	-107	34	4.49	3.3
R V1	30	-107	15	5.2	2.2
L PEa (5)	-53	-32	52	**8.3**	**5.3 (16 vox)**
R PEa (5)	50	-39	49	**7.2**	**5.1 (35 vox)**
L VIP	-24	-58	52	**5.3**	3.2 (2 vox)
R VIP	28	-39	32	4.1	1.44
Subcortical (SARM)					
L IC	-7	-56	2.9	2.5 **(7.6 smooth 3)**	3.5 (smooth at 3)
R IC	10	47	-15	2.7 (4.5 smooth 3)	1.6 (smooth at 3)
L MGN	-20	-42	5.9	2.3 (3.3 smooth 3)	1.16
R MGN	22	-42	7.9	2.3 (3.6 smooth 3)	0.2

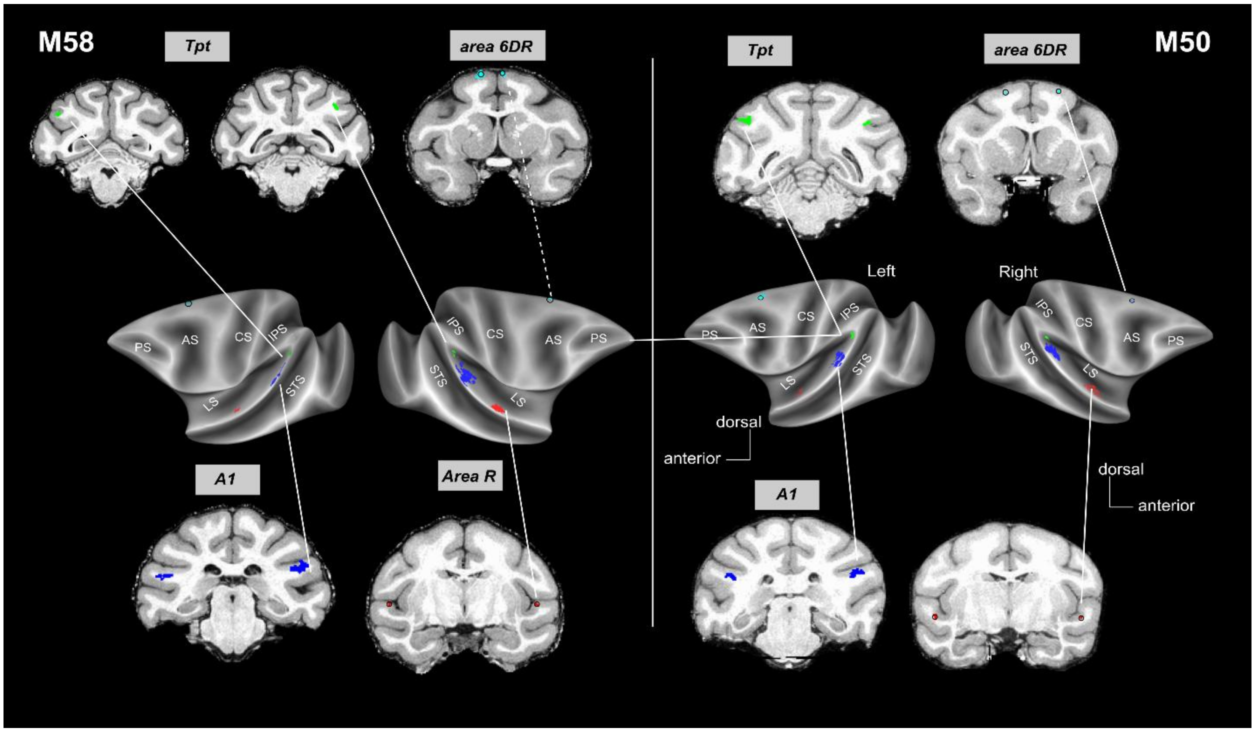

Figure S1: Selected ROIs for analysis in monkey space.

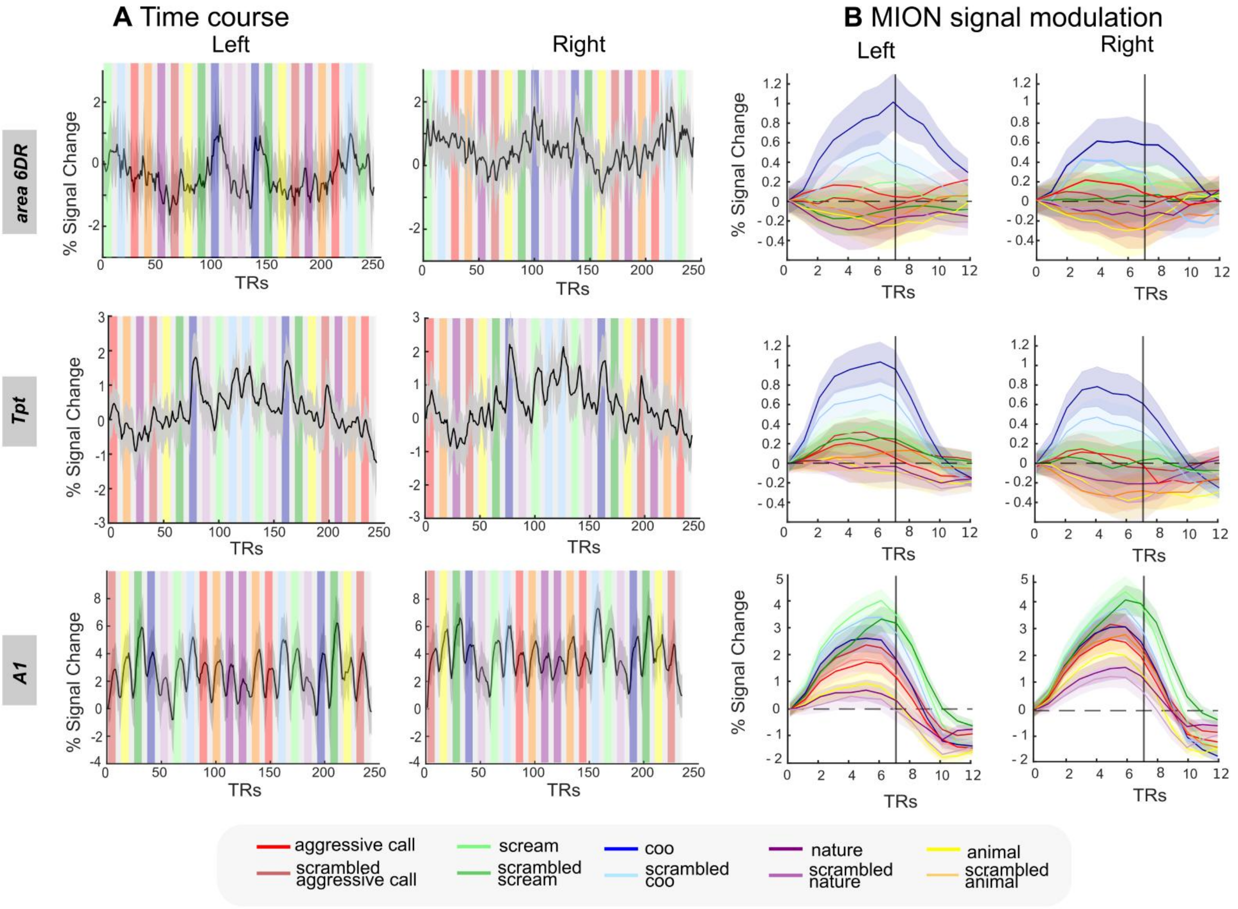

Figure S2: Percent signal change modulation for A1, Tpt and 6DR. (A) Time course (mean of percent signal change +/- SEM) across runs. Note that the time course across the entire run was performed by pooling the runs having the same block organization, thus this plot does not include all the runs that were used for the percent signal change analysis. **(B)** Overall average MION signal modulation from stimulus onset to the end of the baseline block for each condition. Shaded areas represent the standard error of the mean (SEM) across blocks. 0 on the X-axis corresponds to stimulus onset and the vertical black line (TR 7) indicates stimulus offset. To facilitate visual comparison of modulation dynamics from a common origin, each time course condition was normalized by subtracting its respective signal value at the stimulus onset. The y-axis thus reflects the % signal change relative to this adjusted onset value for each condition.

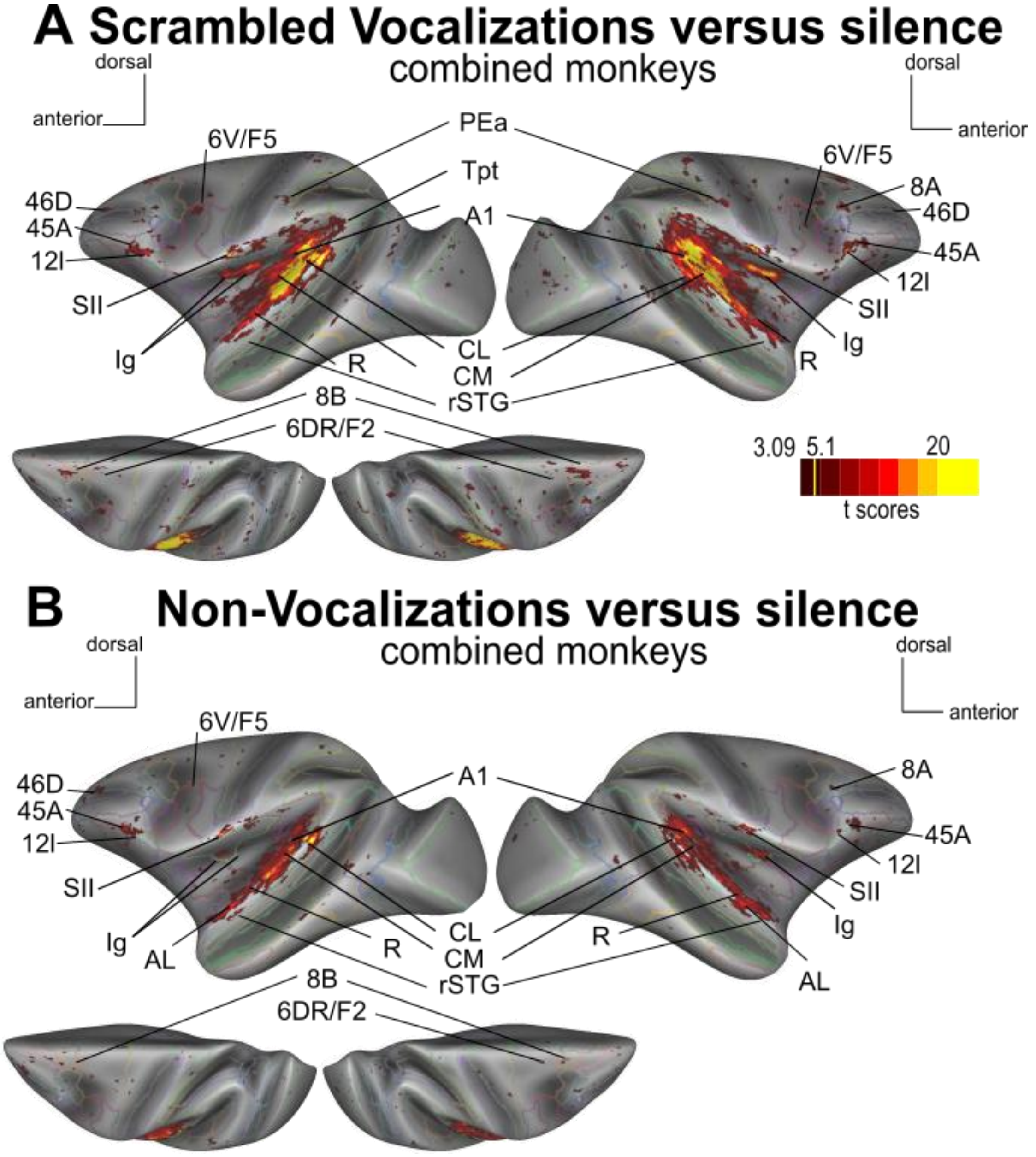

Figure S3 Main vocalization and scrambled vocalization specific cortical activations for the combined monkeys on MEBRAINS template. The SPM t-score map (P<0.001 unc, t-scores> 3.09; P < 0.05 FWE corrected, t-score> 5.1, fixed effect; see color code scale) is projected onto surface representations of the left and right hemispheres of MEBRAINS template, for the contrast vocalization type (aggressive calls; coos and screams) versus silence on the left, and for their scrambled version on the right. Colored lines on the surface represent the delineation of regions according to the CHARM atlas.

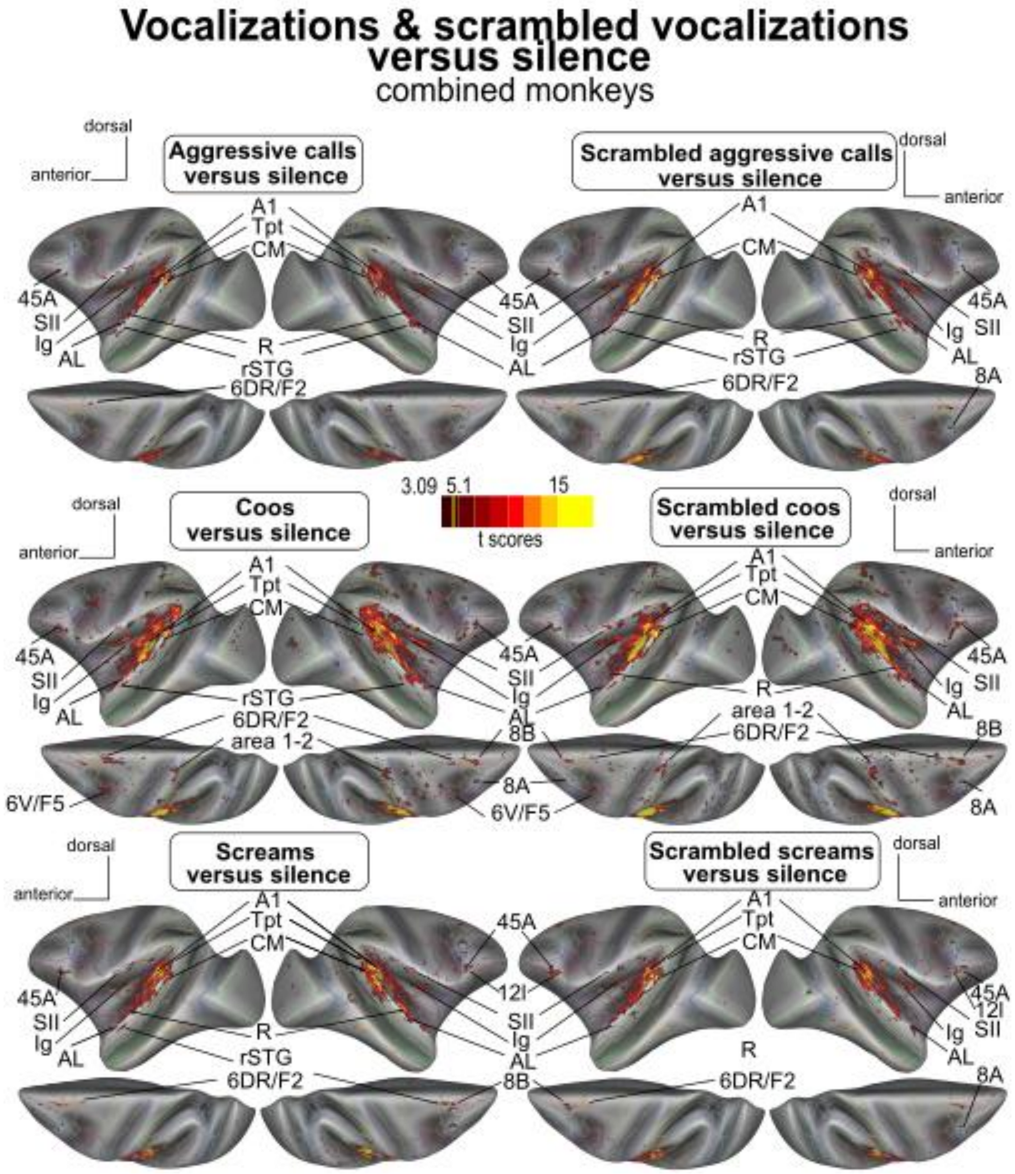

Figure S4 Main vocalization and scrambled vocalization specific cortical activations for the combined monkeys on MEBRAINS template. The SPM t-score map (P<0.001 unc, t-scores> 3.09; P < 0.05 FWE corrected, t-score> 5.1, fixed effect; see color code scale) is projected onto surface representations of the left and right hemispheres of MEBRAINS template, for the contrast vocalization type (aggressive calls, coos and screams) versus silence on the left, and for their scrambled version on the right. Colored lines on the surface represent the delineation of regions according to the CHARM atlas.

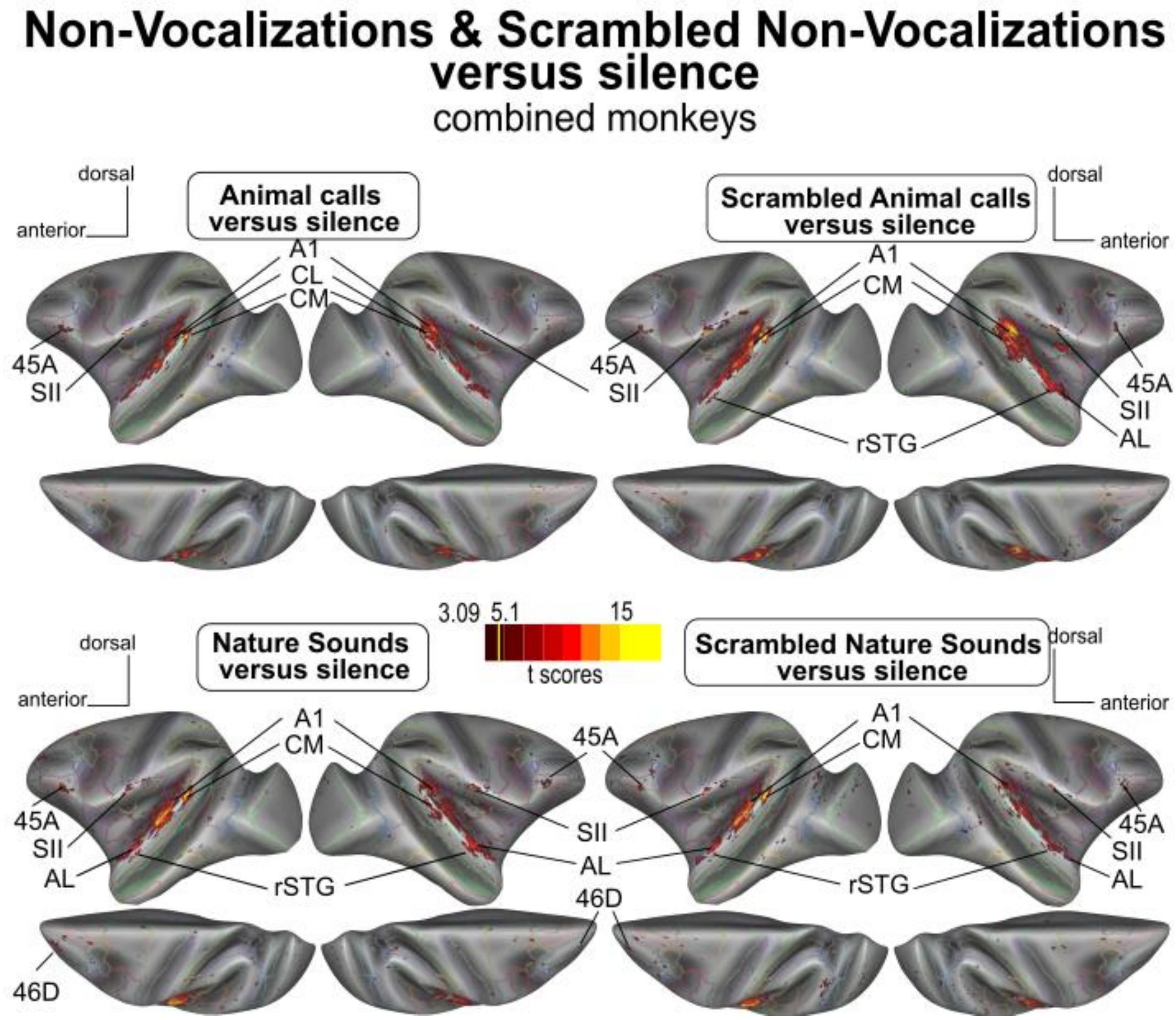

Figure S5 Main non-vocalization and scrambled non-vocalization specific cortical activations for the combined monkeys on MEBRAINS template. The SPM *t*-score map (P<0.001 unc, t-scores> 3.09; *P* < 0.05 FWE corrected, t-score> 5.1, fixed effect; see color code scale) is projected onto surface representations of the left and right hemispheres of MEBRAINS template, for the contrast each vocalization type (animal calls and nature sounds) versus silence on the left and for their scrambled version on the right. Colored lines on the surface represent the delineation of regions according to the CHARM atlas.

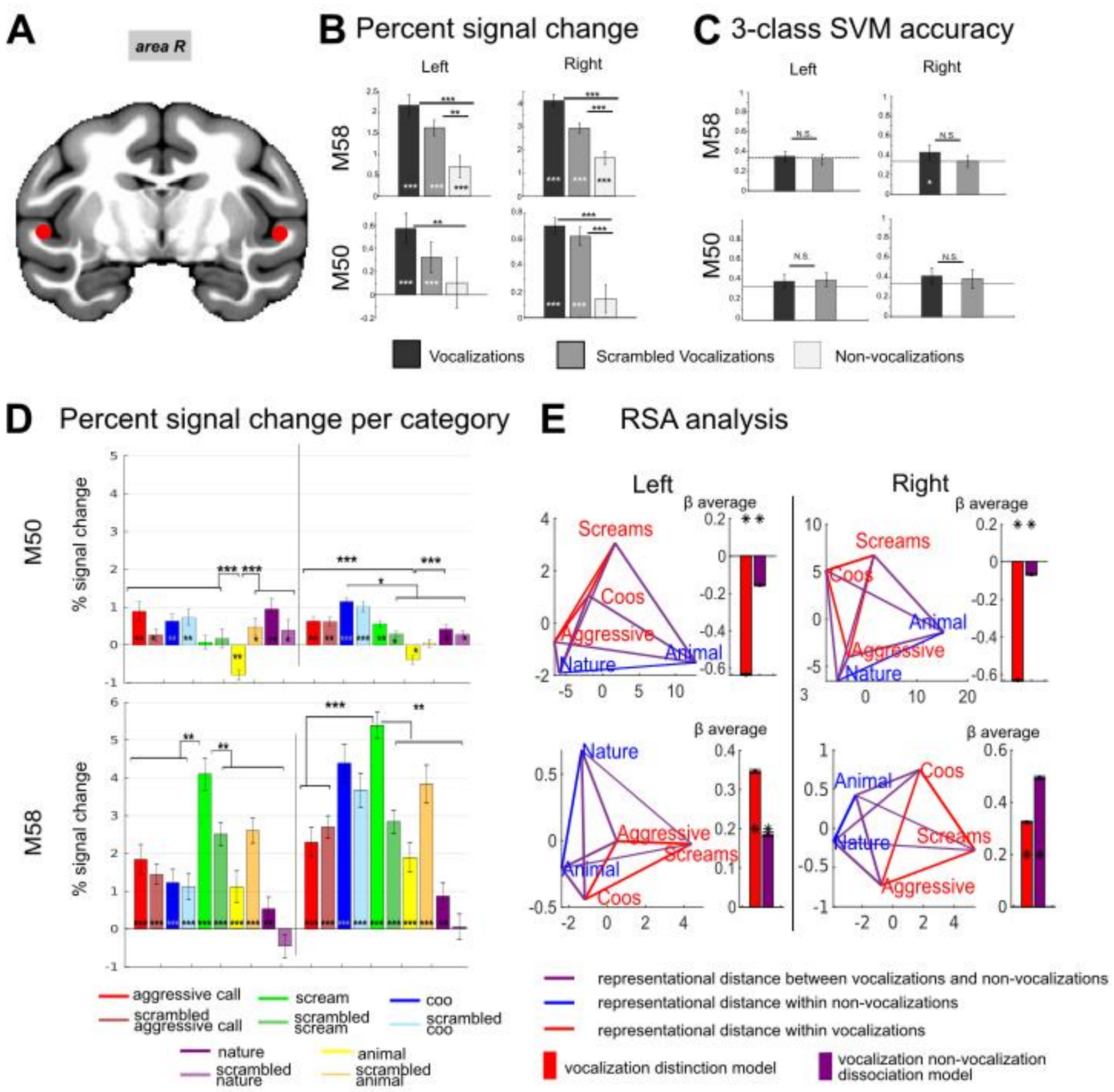

Supplementary Figure S6: Analysis for the ROIs in the rostral core area. A) ROI location. In this figure, ROIs are displayed in the MEBRAINS template (Balan et al., 2023), see figure S2 for the ROIs in monkey space. Precent signal change for B) the vocalizations independent of vocalization type (black), scrambled vocalizations (dark gray), and non-vocalizations (light gray) and each condition D). C) 3-class SVM analysis. For each region of interest (ROI), the accuracies of the 3-class SVM classification for vocalization type (black) and scrambled vocalization type (light gray) are also shown. Comparisons between conditions were performed using the Wilcoxon nonparametric test (***: p<0.001; **: p<0.01; p<0.05). E) Representational geometry of different sound categories for both monkeys. Left side of each panel: 2D visualization of the representational geometry. An average DSM was estimated using a repeated hold-out method and embedded in 2D space through non-classical DSM. The Red/Blue lines indicate distances within the vocalization and non-vocalization categories. The purple lines represent the distance between vocalizations and non-vocalizations. The thickness of the lines corresponds to the normalized similarity in its original space. Right side of each panel: the model fits DSM by a multiple regression RSA, representing the strength of certain coding strategies. The bars show the β average obtained from the repeated hold-out method, with error bar representing confidence intervals. Red bars correspond to the vocalization distinction model where a positive value indicates higher dissimilarity between vocalizations compared to non-vocalizations, and a negative value indicates the opposite. Purple bars are for the vocalizations/non-vocalizations dissociation model, in which a positive value

means a higher dissimilarity between vocalizations and non-vocalizations, than within each group. The stars represent β's significantly different from zero (p<0.05). Results are shown for both M50 and M58.

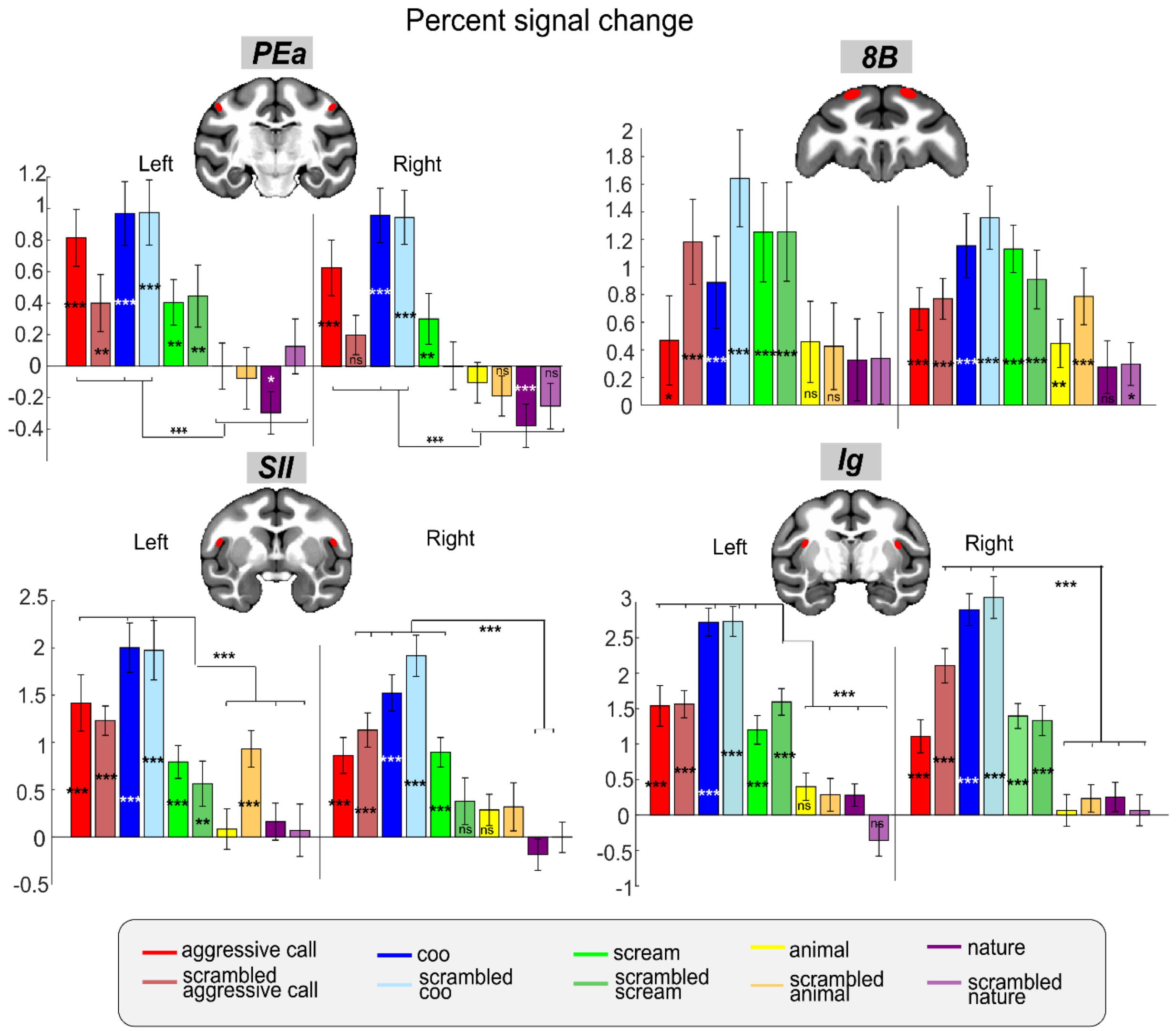

Supplementary Figure S7: Analysis for ROIs: PEa,8B, Ig and SII. Comparisons between conditions were performed using the Wilcoxon nonparametric test (***: p<0.001; **: p<0.01; p<0.05).

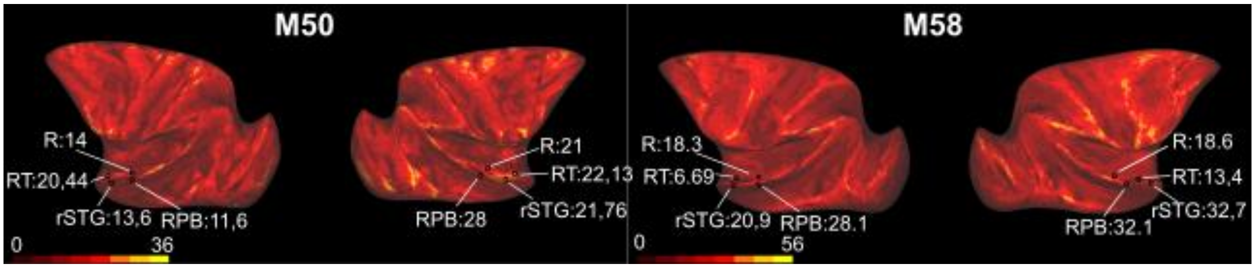

Supplementary Figure S8: temporal signal-to-noise ratio (tSNR; standard deviation divided by mean). TSNR maps are projected on the surface for each subject.

Supplementary table S4: P values for the post-hoc comparisons using the Wilcoxon signed-rank test with Bonferroni correction on the percent signal change for area 6DR. Vocalizations (bold black) and their scrambled version (italic gray/white) are highlighted in dark gray and non-vocalizations (bold black) and their scrambled version (italic gray/white) are highlighted in white. Significant p-values are indicated by the white boxes. For each monkey (M58: upper matrix and M50: lower matrix), the triangle in the upper-right of the matrix represents the p-values of the right hemisphere while the triangle in the lower left of the matrix shows the p-values of the left hemisphere. aggr = aggressive calls.

6DR M58	**Aggr**	***Aggr***	**Coos**	***Coos***	**Screams**	***Screams***	**Animal**	***Animal***	**Nature**	***Nature***
**Aggr**							0.04			0.03
Aggr										
**Coos**	$3x10^{-3}$	0.04			0.01		0.007			0.006
Coos					0.02			0.01		0.01
**Screams**			0.002							
Screams			0.004							
**Animal**			$1.5x10^{-7}$	$4.6x10^{-5}$						
Animal			$5.8x10^{-6}$	0.004						
**Nature**			$3x10^{-7}$	0.003						
Nature		0.01	$1.2x10^{-7}$	$2x10^{-6}$						

6DR M50	**Aggr**	***Aggr***	**Coos**	***Coos***	**Screams**	***Screams***	**Animal**	***Animal***	**Nature**	***Nature***
**Aggr**			$7.9x10^{-5}$							
Aggr			$6x10^{-4}$							
**Coos**	$3.8x10^{-6}$	$6x10^{-4}$				$2.6x10^{-3}$	$8.3x10^{-7}$	$2.4x10^{-7}$	$4x10^{-4}$	$1.2x10^{-5}$
Coos								0.03		
**Screams**			0.04				0.02	0.006		
Screams			$1.3x10^{-7}$	$9.7x10^{-3}$	0.01					
**Animal**			$6.5x10^{-7}$							

Animal			3.8x10^{-6}							
**Nature**			2.5x10^{-7}							
Nature			1.3x10^{-7}	6.8x10^{-3}	0.01					

Supplementary table S5: P values for the post-hoc comparisons using the Wilcoxon signed-rank test with Bonferroni correction on the percent signal change for area Tpt. Same conventions as in Supplementary table S4.

Tpt M58	**Aggr**	*Aggr*	**Coos**	*Coos*	**Screams**	*Screams*	**Animal**	*Animal*	**Nature**	*Nature*
**Aggr**			2.5x10^{-6}	3.9x10^{-4}						
Aggr			0.04			0.01	0.007	0.0011		5.9x10^{-5}
**Coos**	2.2x10^{-7}	9x10^{-4}			2.2x10^{-5}	1.4x10^{-7}	1.3x10^{-7}	1.2x10^{-7}	2.6x10^{-6}	1.2x10^{-7}
Coos	1.8x10^{-3}		0.04		0.002	7.6x10^{-6}	2x10^{-6}	2x10^{-7}	3.9x10^{-4}	1.3x10^{-7}
**Screams**			1.9x10^{-7}	0.00014						
Screams			1.6x10^{-7}	9x10^{-4}						
**Animal**			1.3x10^{-7}	3.9x10^{-4}						
Animal			4.5x10^{-6}	0.02						
**Nature**			6.2x10^{-4}							
Nature		0.029	1.2x10^{-7}	7.2x10^{-7}					0.03	
**Tpt M50**	**Aggr**	*Aggr*	**Coos**	*Coos*	**Screams**	*Screams*	**Animal**	*Animal*	**Nature**	*Nature*
**Aggr**			3x10^{-6}	2.6x10^{-3}						
Aggr			0.03				0.01	0.01	0.04	4.8x10^{-5}
**Coos**	1.8x10^{-7}	6x10^{-4}			2.6x10^{-3}	4.9x10^{-4}	1.3x10^{-7}	6.9x10^{-6}	1.3x10^{-7}	1.2x10^{-7}
Coos	4x10^{-3}						6.5x10^{-6}	3.8x10^{-6}	2.3x10^{-5}	1.3x10^{-7}
**Screams**	0.049		0.04							6x10^{-4}
Screams			0.001							6x10^{-4}
**Animal**		0.042	1.2x10^{-7}	3.8x10^{-6}	1.6x10^{-4}	0.01				
Animal			1.2x10^{-7}	2.9x10^{-5}	9.4x10^{-4}					
**Nature**			1.2x10^{-7}	6.5x10^{-6}	2.5x10^{-4}	0.02				
Nature			1.2x10^{-7}	2.9x10^{-5}	9.4x10^{-4}					

Supplementary table S6: P values for the post-hoc comparisons using the Wilcoxon signed-rank test with Bonferroni correction on the percent signal change for area A1. Same conventions as in Supplementary table S4.

A1 M58	Aggr	*Aggr*	Coos	*Coos*	Screams	*Screams*	Animal	*Animal*	Nature	*Nature*
Aggr				0.004	$4.6x10^{-7}$					0.02
Aggr					$7.7x10^{-4}$				$4.9x10^{-3}$	$3.6x10^{-5}$
Coos	0.0059						0.034		$2x10^{-6}$	$1.3x10^{-7}$
Coos	0.004						$2.2x10^{-3}$		$1.4x10^{-7}$	$1.3x10^{-7}$
Screams	$5.8x10^{-6}$					$1.9x10^{-3}$	$2.6x10^{-7}$	$8.6x10^{-4}$	$1.3x10^{-7}$	$1.3x10^{-7}$
Screams	0.039								$6.2x10^{-4}$	$2.6x10^{-6}$
Animal			0.034	0.02	$7.6x10^{-5}$					0.034
Animal					0.01				$3.9x10^{-4}$	$1.5x10^{-6}$
Nature		$1x10^{-4}$	$2.2x10^{-7}$	$1.7x10^{-7}$	$1.2x10^{-7}$	$2.6x10^{-6}$		0.004		
Nature		$9x10^{-5}$	$1.9x10^{-7}$	$1.6x10^{-7}$	$1.2x10^{-7}$	$2x10^{-6}$		0.003		
A1 M50	Aggr	*Aggr*	Coos	*Coos*	Screams	*Screams*	Animal	*Animal*	Nature	*Nature*
Aggr			$7.9x10^{-5}$		$1.3x10^{-6}$					
Aggr					0.03		$6.1x10^{-4}$		0.049	$1.8x10^{-5}$
Coos	$3.8x10^{-5}$	0.03					$1.2x10^{-7}$		$6.5x10^{-7}$	$1.26x10^{-7}$
Coos	0.01							$2.3x10^{-5}$	$4.6x10^{-3}$	$5.26x10^{-7}$
Screams	$3.9x10^{-4}$						$1.2x10^{-7}$	0.02	$1.3x10^{-7}$	$1.26x10^{-7}$
Screams							$8.4x10^{-6}$		0.002	$2.4x10^{-7}$
Animal		0.009	$1.3x10^{-7}$	$1.4x10^{-6}$	$1.3x10^{-7}$			0.001		
Animal			0.0017		0.01					$3.8x10^{-5}$
Nature			$3.8x10^{-6}$	0.0026						
Nature		$5x10^{-4}$	$1.2x10^{-7}$	$1.4x10^{-7}$	$3.8x10^{-5}$			0.01		

Supplementary table S7: P values for the post-hoc comparisons using the Wilcoxon signed-rank test with Bonferroni correction on the percent signal change for area R. Same conventions as in Supplementary table S4.

TVa M58	Aggr	*Aggr*	Coos	*Coos*	Screams	*Screams*	Animal	*Animal*	Nature	*Nature*
Aggr					$3.9x10^{-4}$					0.039
Aggr					$3.9x10^{-4}$					0.039
Coos									$7.6x10^{-5}$	$5.8x10^{-6}$

Coos									$9.7x10^{-5}$	$7.6x10^{-6}$
**Screams**	0.014	0.017	$2.2x10^{-3}$	$4.9x10^{-3}$		$4x10^{-4}$	$7.7x10^{-4}$		$1.2x10^{-7}$	$1.2x10^{-7}$
Screams									0.034	$5.9x10^{-3}$
**Animal**					$3.6x10^{-5}$					0.02
Animal							0.015		$2.2x10^{-3}$	$2.5x10^{-3}$
**Nature**					$4.5x10^{-6}$	0.029		$3.3x10^{-3}$		
Nature	0.034	0.029			$1.2x10^{-7}$	$5.9x10^{-5}$		$2.6x10^{-6}$		
**TVa** M50	**Aggr**	***Aggr***	**Coos**	***Coos***	**Screams**	***Screams***	**Animal**	***Animal***	**Nature**	***Nature***
**Aggr**							$1.8x10^{-5}$			
Aggr							$7.6x10^{-4}$			
**Coos**						$1.7x10^{-4}$	$1.2x10^{-7}$	$2.3x10^{-5}$	0.049	$6.1x10^{-4}$
Coos						$5.6x10^{-4}$	$1.2x10^{-7}$	$1x10^{-4}$		0.002
**Screams**							0.001			
Screams							0.049			
**Animal**	$6.5x10^{-7}$	0.042	$2.5x10^{-4}$	$8.4x10^{-6}$	0.042	0.026			0.0017	
Animal							$1.4x10^{-3}$			
**Nature**							$2.9x10^{-6}$			
Nature							0.019			

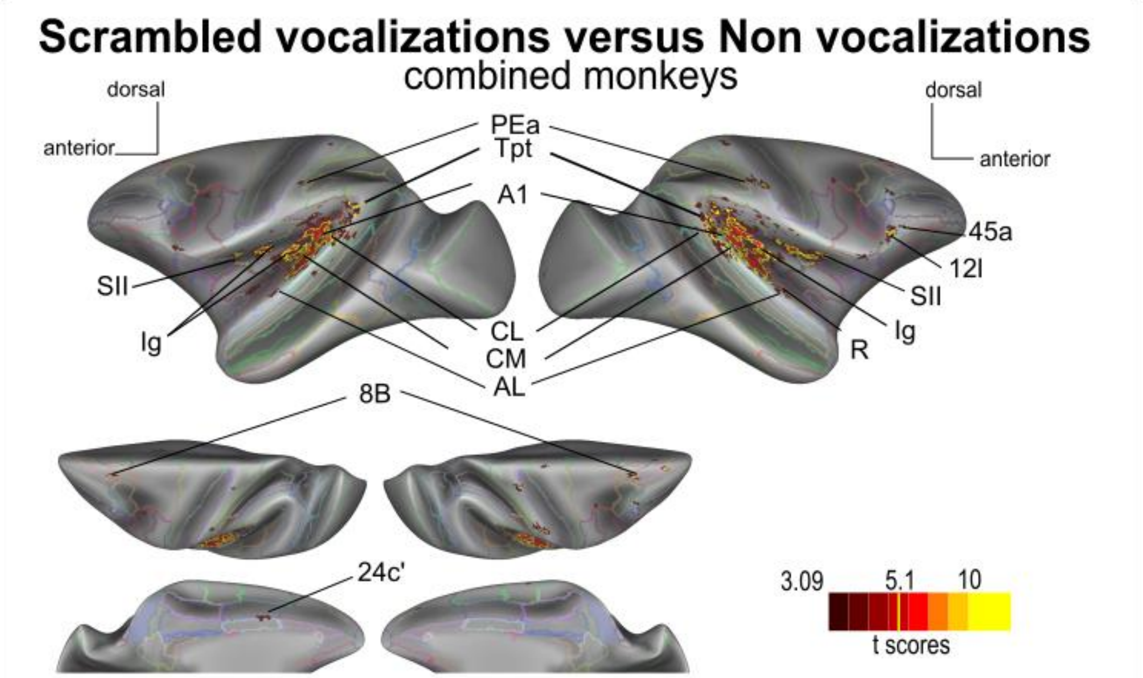

Figure S9 Main auditory cortical activations for the contrast "scrambled vocalizations" versus "non vocalizations" for the monkeys combined (M58 and M50). Projection of SPM *t*-score maps (P<0.001 uncorrected, t > 3.09; *P* < 0.05 FWE corrected, t-score> 5.1 outlined in yellow, fixed effect - see color scale bar; 10 voxels cluster-level threshold of p < 0.05 False Discovery Rate (FDR)-corrected) onto surface representations of the left and right hemispheres of the MEBRAINS template (Balan et al., 2023).
